# SlWRKY30 and SlWRKY81 synergistically modulate tomato immunity to *Ralstonia solanacearum* by directly regulating *SlPR-STH2*

**DOI:** 10.1093/hr/uhad050

**Published:** 2023-03-15

**Authors:** Fengfeng Dang, Jinhui Lin, Yajing Li, Ruoyun Jiang, Yudong Fang, Fei Ding, Shuilin He, Yanfeng Wang

**Affiliations:** State Key Laboratory for Conservation and Utilization of Subtropical Agro­Bioresources, College of Life Sciences, South China Agricultural University, Guangzhou 510642, China; Shaanxi Key Laboratory of Chinese Jujube, Yan’an University, Yan’an, Shaanxi 716000, China; College of Agriculture, Fujian Agriculture and Forestry University, Fuzhou 350002, China; State Key Laboratory for Conservation and Utilization of Subtropical Agro­Bioresources, College of Life Sciences, South China Agricultural University, Guangzhou 510642, China; College of Horticulture, Fujian Agriculture and Forestry University, Fuzhou 350002, China; State Key Laboratory for Conservation and Utilization of Subtropical Agro­Bioresources, College of Life Sciences, South China Agricultural University, Guangzhou 510642, China; School of Life Sciences, Liaocheng University, Liaocheng 252000, China; College of Agriculture, Fujian Agriculture and Forestry University, Fuzhou 350002, China; Shaanxi Key Laboratory of Chinese Jujube, Yan’an University, Yan’an, Shaanxi 716000, China

## Abstract

Bacterial wilt is a devastating disease of tomato (*Solanum lycopersicum*) caused by *Ralstonia solanacearum* that severely threatens tomato production. Group III WRKY transcription factors (TFs) are implicated in the plant response to pathogen infection; however, their roles in the response of tomato to *R. solanacearum* infection (RSI) remain largely unexplored. Here, we report the crucial role of SlWRKY30, a group III SlWRKY TF, in the regulation of tomato response to RSI. *SlWRKY30* was strongly induced by RSI. *SlWRKY30* overexpression reduced tomato susceptibility to RSI, and also increased H_2_O_2_ accumulation and cell necrosis, suggesting that SlWRKY30 positively regulates tomato resistance to RSI. RNA sequencing and reverse transcription–quantitative PCR revealed that *SlWRKY30* overexpression significantly upregulated pathogenesis-related protein (*SlPR-STH2*) genes *SlPR­STH2a*, *SlPR­STH2b*, *SlPR­STH2c*, and *SlPR­STH2d* (hereafter *SlPR­STH2a*/*b*/*c*/*d*) in tomato, and these *SlPR-STH2* genes were directly targeted by SlWRKY30. Moreover, four group III WRKY proteins (SlWRKY52, SlWRKY59, SlWRKY80, and SlWRKY81) interacted with SlWRKY30, and *SlWRKY81* silencing increased tomato susceptibility to RSI. Both SlWRKY30 and SlWRKY81 activated *SlPR­STH2a*/*b*/*c*/*d* expression by directly binding to their promoters. Taking these results together, SlWRKY30 and SlWRKY81 synergistically regulate resistance to RSI by activating *SlPR-STH2a*/*b*/*c*/*d* expression in tomato. Our results also highlight the potential of *SlWRKY30* to improve tomato resistance to RSI via genetic manipulations.

## Introduction

To defend against pathogen attack, plants have evolved sophisticated innate immune systems, which including pattern-triggered immunity (PTI) and effector-triggered immunity (ETI) [1, 2]. Despite the distinct dynamics in ETI and PTI, they share common signaling components, such as Ca^2+^, various kinases, salicylic acid (SA), jasmonic acid (JA), and ethylene [3, 4]. The immune signals accumulate in the nucleus, where they are integrated and translated by various transcription factors (TFs) to promote massive transcriptional reprogramming, thereby activating PTI and ETI [5]. As the transcription of immunity-related genes is largely determined by specific targeting of various TFs, these TFs are crucial for proper regulation of plant immunity to pathogens [6]. Several TF families, e.g. APETALA2 (AP2)/ethylene response factor (ERF), basic leucine zipper (bZIP)/TGACG motif-binding proteins (TGAs), basic helix–loop–helix (bHLH), WRKY, and NAC, control the activation of plant immunity [7, 8].

WRKY TFs comprise one of the largest TF families; its members contain at least one conserved WRKY domain, which is a DNA-binding domain that preferably binds to the W­box motif (C/T)TGAC(C/T) in the promoter region of their target genes. WRKY TFs are divided into three distinct groups (I, II, and III) based on the number of WRKY domains and the amino acid sequence features of zinc-finger motifs [9]. WRKY TFs are involved in the regulation of plant immunity; in particular, the group III WRKY TFs positively or negatively modulate plant innate immunity [10, 11]. In *Arabidopsis* (*Arabidopsis thaliana*), SA is sensed by two different classes of receptors, NONEXPRESSER OF PR GENES 1 (NPR1) and its homologs NPR3/NPR4, which function independently to modulate SA­mediated immunity through regulating *WRKY70* (group III) expression [12, 13]. During ETI, the group III WRKY54 and WRKY70, which are substrates of the NPR1-cullin-RING ligase 3 (CRL3) complex, promote SA-mediated cell survival [14]. Moreover, many WRKY members from different plant species are transcriptionally modified in response to pathogen attack; some positively [15, 16] and others negatively [17] regulate plant immunity. Further, some WRKY TFs interact with each other at the transcriptional or post-translational level [16]. These functionally connected WRKY TFs may form transcriptional networks, with some having central roles in mediating rapid and efficient activation of host defense programs [18]. Nevertheless, how TFs are organized into networks and how they operate remain unclear.

**Figure 1:**
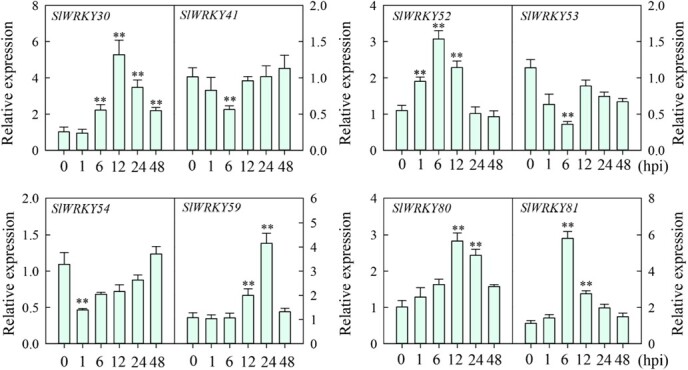
Expression analysis of the eight group III *SlWRKY* genes induced by RSI in tomato*.* Expression levels of the eight group III *SlWRKY* genes in tomato leaves analyzed by RT–qPCR from 0 to 48 hpi with *R. solanacearum*. Relative expression of the genes in *R. solanacearum*-inoculated plants was compared with the expression in control plants, which was set to 1. Data represent the mean ± standard error.

Tomato (*Solanum lycopersicum*) is a major horticultural crop that has been adapted to a wide range of environmental conditions worldwide. Bacterial wilt, one of the most devastating soil-borne diseases, is caused by *Ralstonia solanacearum*, which constrains the production and geographical distribution of tomato [19, 20]. Therefore, understanding how tomato responds to *R. solanacearum* infection (RSI) would facilitate the development of effective strategies to control this disease. Around 81–83 WRKY TFs are encoded in the tomato genome [21, 22], among which SlWRKY3, SlWRKY31, SlWRKY33, SlWRKY39, SlWRKY45, SlWRKY72, SlWRKY73, SlWRKY74, and SlWRKY70 [22–24] function in plant immunity to biotic stresses, and SlWRKY23, SlWRKY46, SlWRKY53/SlWRKY54, SlWRKY80, and SlWRKY81 are transcriptionally modified during pathogen attack [22]. However, no SlWRKY TF has been implicated in the tomato response to RSI.

In this article, we investigated the behavior of the group III WRKY TFs during RSI and revealed that SlWRKY30 positively regulates tomato resistance to RSI. *SlWRKY30* was strongly induced during RSI in tomato, and *SlWRKY30-*overexpressing tomato plants had increased resistance to RSI. Transcriptome analysis revealed that SlWRKY30 transcriptionally activates defense-response genes. In particular, expression of the pathogenesis-related protein (*SlPR-STH2*) genes *SlPR-STH2a*, *SlPR-STH2b*, *SlPR­STH2c*, and *SlPR-STH2d* (hereafter *SlPR-STH2a*/*b*/*c*/*d*) was upregulated by SlWRKY30, which directly bound to the W-boxes in the *SlPR­STH2a*/*b*/*c*/*d* promoters. Furthermore, SlWRKY30 interacted with SlWRKY81 to synergistically activate *SlPR­STH2a*/*b*/*c*/*d* expression. Therefore, we revealed a genetic resource for breeding bacterial wilt­resistant tomato varieties.

## Results

### Expression analysis of group III *SlWRKY* genes during *R. solanacearum* infection and exogenous application of salicylic acid

To identify group III *SlWRKY* genes potentially involved in the tomato response to RSI, we analyzed their transcriptional response to RSI by reverse transcription–quantitative PCR (RT–qPCR). Expression of *SlWRKY30*, *52*, *59*, *80*, and *81* was significantly upregulated following RSI. In particular, expression of *SlWRKY30* [12 hours post-inoculation (hpi)] and *SlWRKY81* (6 hpi) was 5.2- and 5.8-fold higher than that of the other genes, respectively (Fig. 1).

Since SA is a crucial signaling molecule that is involved in plant defense against biotrophic [25] and hemibiotrophic pathogens, such as *R. solanacearum* [26], we therefore analyzed the response of the group III *SlWRKY* genes to exogenous SA application. *SlWRKY30*, *52*, *59*, *80*, and *81* were upregulated by SA, similar to their response to RSI, while *SlWRKY41*, *SlWRKY53*, and *SlWRKY54* were downregulated (Supplementary Data Fig. S1A). Next, we analyzed the expression of these *SlWRKY* genes in different tissues (stem, leaf, and root) under normal conditions using RT–qPCR and observed similar expression patterns (except for *SlWRKY54*), with relatively higher expression in the leaf and root than in the stem. Conversely, *SlWRKY54* expression was higher in the stem than in the leaf and root (Supplementary Data Fig. S1B). Taken together, these results indicate that these eight group III SlWRKY TFs are involved in tomato immunity to RSI.

### Subcellular localization and transcriptional activity of the eight group III SlWRKY transcription factors

**Figure 2:**
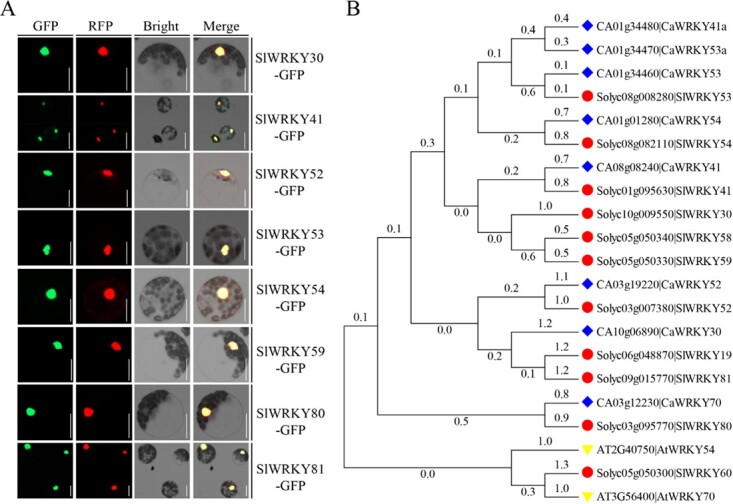
Characterization of the group III SlWRKY members. (A) Subcellular localization of SlWRKY–GFP fusion proteins; we transiently expressed the different SlWRKY–GFP proteins in *Arabidopsis* mesophyll protoplasts. Protoplasts were incubated in WI buffer for 10 hours after transformation and imaged using a fluorescence microscope. Scale bar = 20 μm. (B) Phylogenetic tree of group III WRKY TFs from tomato, pepper, and *Arabidopsis*. Amino acid sequences labeled with red circles, dark blue diamonds, and inverted yellow triangles represent the group III WRKY TFs from tomato, pepper and *Arabidopsis*, respectively. The tree was constructed using MEGA 6.06.

The biological function of a TF is closely associated with its subcellular localization. We therefore investigated the subcellular localization of these eight group III SlWRKY TFs through transiently expressed SlWRKY–GFP (green fluorescent protein) fusion proteins in *Arabidopsis* mesophyll protoplasts. All eight SlWRKY–GFP fusion proteins localized exclusively to the nuclei, with a complete overlap with the red fluorescent protein (RFP) nuclear marker protein, indicating that these SlWRKY TFs function in the nucleus (Fig. 2A, Supplementary Data Fig. S2A and B). Next, we analyzed the transcriptional activity of these SlWRKY proteins by a transcriptional activation assay in yeast strain AH109. The BD–SlWRKY fusions activated *LacZ* reporter gene expression (Supplementary Data Fig. S3A), indicating that these SlWRKY TFs have transcriptional activation activity.

Furthermore, we conducted a phylogenetic analysis to investigate the relationship among the group III SlWRKYs and their orthologs in pepper (*Capsicum annuum*), and constructed two unrooted phylogenetic trees based on amino acid sequence similarities using the neighbor-joining method. SlWRKY30 shared highest sequence similarity with CaWRKY41 (Fig. 2B, Supplementary Data Fig. S3B), which confers pepper resistance to RSI [42]. Together, these results indicate the eight group III SlWRKY members are TFs and may function in the tomato response to RSI.

### SlWRKY30 positively regulates tomato resistance to *R. solanacearum* infection

**Figure 3:**
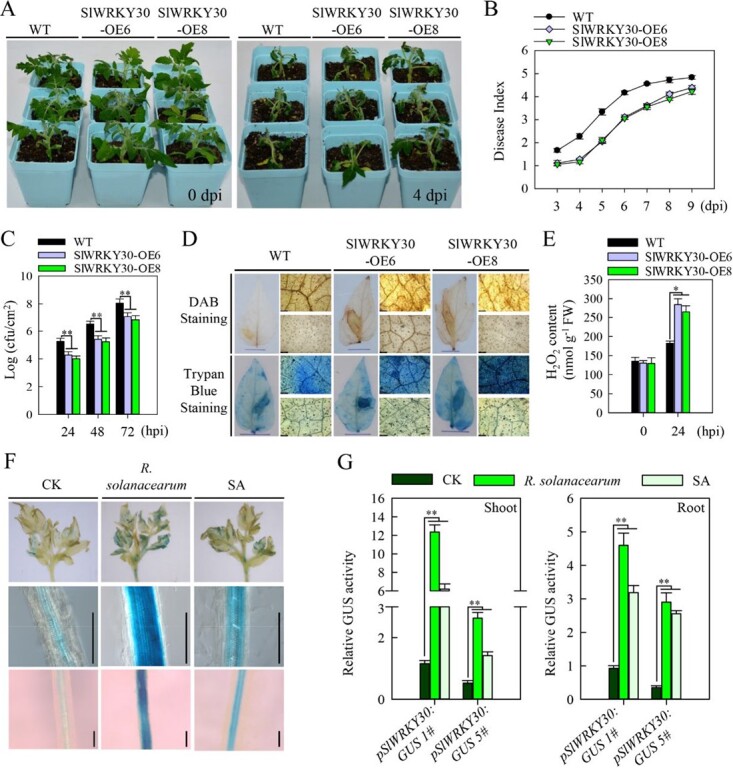
*SlWRKY30* overexpression enhances tomato resistance to RSI. (A) Resistance levels in WT, *SlWRKY30-OE6*, and *SlWRKY30-OE8* tomato plants at 0 and 4 dpi with *R. solanacearum*. (B) *R. solanacearum-*infected WT, *SlWRKY30-OE6*, and *SlWRKY30-OE8* plants were scored daily using a disease index. (C) Bacterial growth in WT, *SlWRKY30-OE6*, and *SlWRKY30-OE8* leaves following RSI*.* (D) Increased H_2_O_2_ levels and cell death in *SlWRKY30-OE6* and *SlWRKY30-OE8* leaves compared with the WT at 24 hpi with *R. solanacearum*. (E) H_2_O_2_ concentration in WT, *SlWRKY30-OE6*, and *SlWRKY30-OE8* leaves. (F) GUS expression in *pSlWRKY30:GUS* tomato shoots and roots that were infected with *R. solanacearum* or received exogenous application of SA for 24 h. Scale bar = 150 μm. (G) GUS activity in *pSlWRKY30:GUS* tomato shoots and roots that were infected with *R. solanacearum* or received exogenous application of SA for 24 h. Relative GUS activity in treated plants was compared with activity in control plants, which was set to 1. Data represent the mean ± standard error.

Among the group III *SlWRKY* genes, *SlWRKY30* was upregulated by RSI and SA treatment in tomato. *SlWRKY30* was also induced by JA and ACC (the precursor of ethylene) (Supplementary Data Fig. S4A). Therefore, we considered *SlWRKY30* a strong candidate gene for a role in the tomato immune response. To study the role of SlWRKY30 in tomato immunity to RSI, we generated nine independent *SlWRKY30­*overexpressing *T*_2_ or *T*_3_ tomato transgenic lines, which had higher *SlWRKY30* expression levels compared with wild-type (WT) plants (Supplementary Data Fig. S4B). We selected two independent lines (OE6 and OE8) for an RSI assay. OE6 and OE8 exhibited similar growth and development phenotypes to WT plants under normal conditions. Following RSI, all of the tomato plants exhibited wilt symptoms (Fig. 3A), but the *SlWRKY30*­overexpressing lines showed decreased susceptibility compared with WT plants at 4 days post-inoculation (dpi), as evidenced by a lower disease index and growth rate of *R. solanacearum* (Fig. 3B and C).

RSI induced H_2_O_2_ accumulation and cell death in both the WT and transgenic plants, but we detected more H_2_O_2_ accumulation and heavier cell death in OE6 and OE8 than in the WT at 24 hpi (Fig. 3D and E). To verify these results, we silenced *SlWRKY30* in tomato plants via virus-induced gene silencing (VIGS) with the *TRV:Slwrky30* construct. Compared with control plants (infected with the *TRV:00* empty vector), *SlWRKY30*­silenced plants had increased susceptibility to RSI at 4 dpi (Supplementary Data Fig. S5A and B), indicated by a higher disease index and *R. solanacearum* growth rate (Supplementary Data Fig. S5C and D). Together, these results demonstrate that SlWRKY30 positively modulates tomato resistance to RSI.

*R. solanacearum* exclusively invades plant roots [19, 20], colonizes the root cortex, and spreads through xylem vessels to systemically infect the whole plant [27]. To test if *SlWRKY30* is expressed in vascular tissues to induce an immune response, we generated *pSlWRKY30:GUS* reporter lines in tomato and analyzed the *SlWRKY30* tissue-specific expression pattern in *T*_2_ plants upon RSI or SA treatment. Under normal conditions, we observed β­glucuronidase (GUS) activity mainly in the leaf veins and root stele but not in the cortex, while both RSI and SA significantly promoted GUS activity (Fig. 3F and G; Supplementary Data Fig. S4C), indicating that RSI and SA regulate *SlWRKY30* transcription.

### SlWRKY30 functions in tomato immunity to *R. solanacearum* infection by directly regulating *SlPR­STH2*

**Figure 4:**
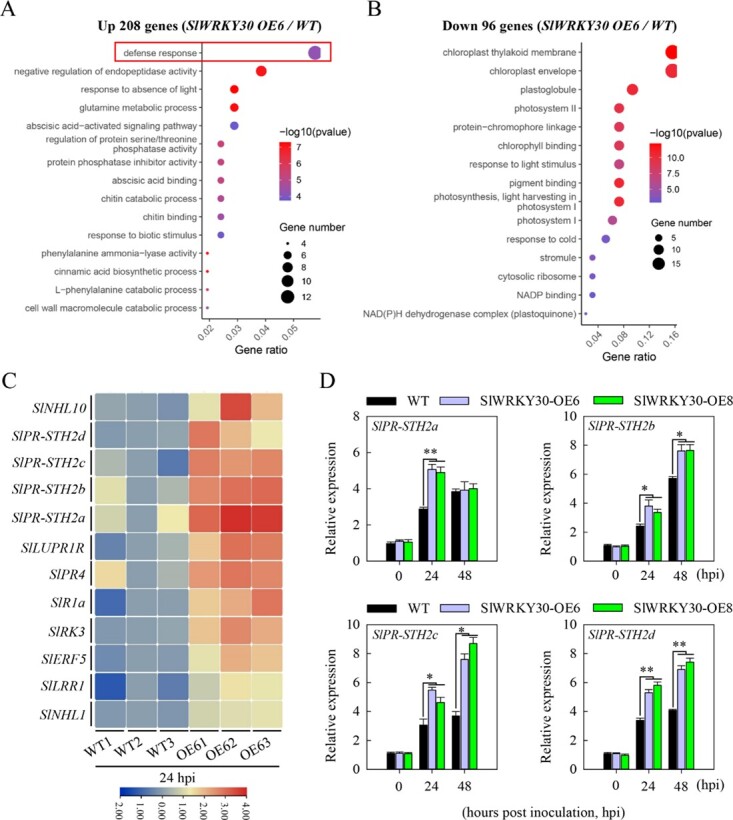
Transcriptome profiling identified *R. solanacearum*-induced SlWRKY30-regulated genes in tomato. (A, B) GO enrichment analysis showing that diverse terms are enriched among the DEGs regulated by SlWRKY30 in tomato at 24 hpi with *R. solanacearum*. (C) Heat map showing that SlWRKY30 upregulated defense-related genes in tomato at 24 hpi with *R. solanacearum*. The color bar indicates the log_2_ fold change. (D) Expression levels of *SlPR-STH2a*, *SlPR-STH2b*, *SlPR-STH2c*, and *SlPR-STH2d* analyzed by RT–qPCR in WT, *SlWRKY30-OE6*, and *SlWRKY30-OE8* tomato plants at 0, 24, and 48 hpi with *R. solanacearum*. Data represent the mean ± standard error.

To identify genes regulated by SlWRKY30 during the tomato response to RSI, we compared the transcriptome profiles of the OE6 line and WT plants at 24 hpi with *R. solanacearum* by transcriptome sequencing (RNA-seq). We identified the differentially expressed genes (DEGs) and filtered them based on the criteria of fold change ≥2 and false discovery rate <.01. The expression of 304 genes was significantly affected, among which 208 were upregulated and 96 were downregulated (Fig. 4A and B, Supplementary Data Table S2). The most enriched term in Gene Ontology (GO) enrichment analysis was ‘defense response’, which involved 12 genes (Fig. 4A, Supplementary Data Table S3), all of which had increased expression in OE6 (Fig. 4C).

Among these defense-related genes were the pathogenesis-related protein (*SlPR-STH2*) genes *SlPR­STH2a* (Solyc09g090970), *SlPR-STH2b* (Solyc09g090980), *SlPR-STH2c* (Solyc09g090990), and *SlPR-STH2d* (Solyc09g091000), which are closely related and share ~70% identity in their deduced amino acid sequences (Supplementary Data Fig. S6A). In addition, these *SlPR-STH2* genes are highly conserved among different plant species and are generally induced by pathogen attack [26, 28–30], implying that SlWRKY30 positively regulates tomato immunity to RSI by modulating *SlPR-STH2a*/*b*/*c*/*d*. To test this, we compared *SlPR­STH2a*/*b*/*c*/*d* expression between the *SlWRKY30-*overexpressing lines (OE6 and OE8) and WT plants upon RSI by RT–qPCR. *SlPR-STH2a*/*b*/*c*/*d* expression was markedly increased in *SlWRKY30­*overexpressing lines compared with WT plants following RSI (Fig. 4D).

**Figure 5:**
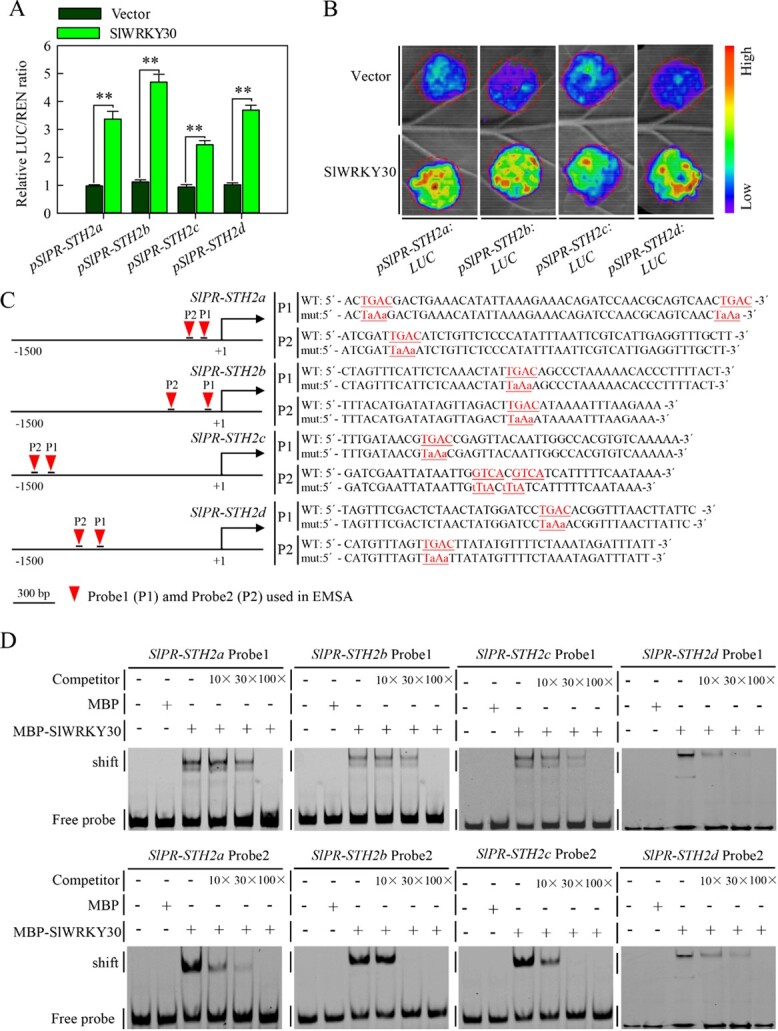
SlWRKY30 directly activates *SlPR-STH2a*, *SlPR-STH2b*, *SlPR-STH2c*, and *SlPR­STH2d* expression in tomato. (A) Dual-luciferase assay showing that SlWRKY30 activates the expression of the *LUC* reporter gene driven by *SlPR-STH2a*/*b*/*c*/*d* promoters. The LUC/REN ratio represents the relative activity of the *SlPR­STH2a*/*b*/*c*/*d* promoters. Values represent mean ± standard error. (B) Transient expression assay showing that SlWRKY30 transcriptionally activates the *LUC* reporter gene (driven by the *SlPR-STH2a*/*b*/*c*/*d* promoters). (C) Schematic diagrams of promoter sequence selection for the EMSA. The red triangles indicate the sequence position used for the EMSA. For the W-box mutants, the respective W-box (TGAC) was mutated to TaAa. (D) EMSA showing that SlWRKY30 directly binds to the *SlPR-STH2a*/*b*/*c*/*d* promoters.

Next, we performed transient expression assays in *Nicotiana benthamiana* leaves using dual­luciferase reporter plasmids (Supplementary Data Fig. S6B). Compared with the empty vector (*p62-SK*) control, co-expression of *SlWRKY30* driven by the *CaMV35S* promoter with *LUC* driven by the *SlPR­STH2a*/*b*/*c*/*d* promoters enhanced *LUC* reporter gene expression and LUC luminescence intensity (Fig. 5A and B). Furthermore, we identified W-boxes, which are preferentially bound by WRKY TFs [9], in the *SlPR­STH2a*/*b*/*c*/*d* promoters (Fig. 5C), implying that these *SlPR-STH2* genes are directly activated by SlWRKY30. To test this, we performed an electrophoretic mobility shift assay (EMSA) using MBP (maltose-binding protein)–WRKY30 fusion protein and DNA probes (WT and mutant) designed from the *SlPR-STH2a*/*b*/*c*/*d* promoters containing the W-box. MBP–SlWRKY30, but not MBP alone, directly bound to DNA probes 1 and 2 (WT), were repressed by the competitors (Fig. 5D). Moreover, SlWRKY30 does not bind to DNA probes, in which the TGAC motifs were mutated to TaAa (Supplementary Data Fig. S6C). Together, these results indicate that SlWRKY30 positively regulates tomato immunity to RSI by directly activating *SlPR­STH2a*/*b*/*c*/*d* expression.

### SlWRKY30 interacts with SlWRKY52, 59, 80, and 81 during the response to *R. solanacearum* infection

**Figure 6:**
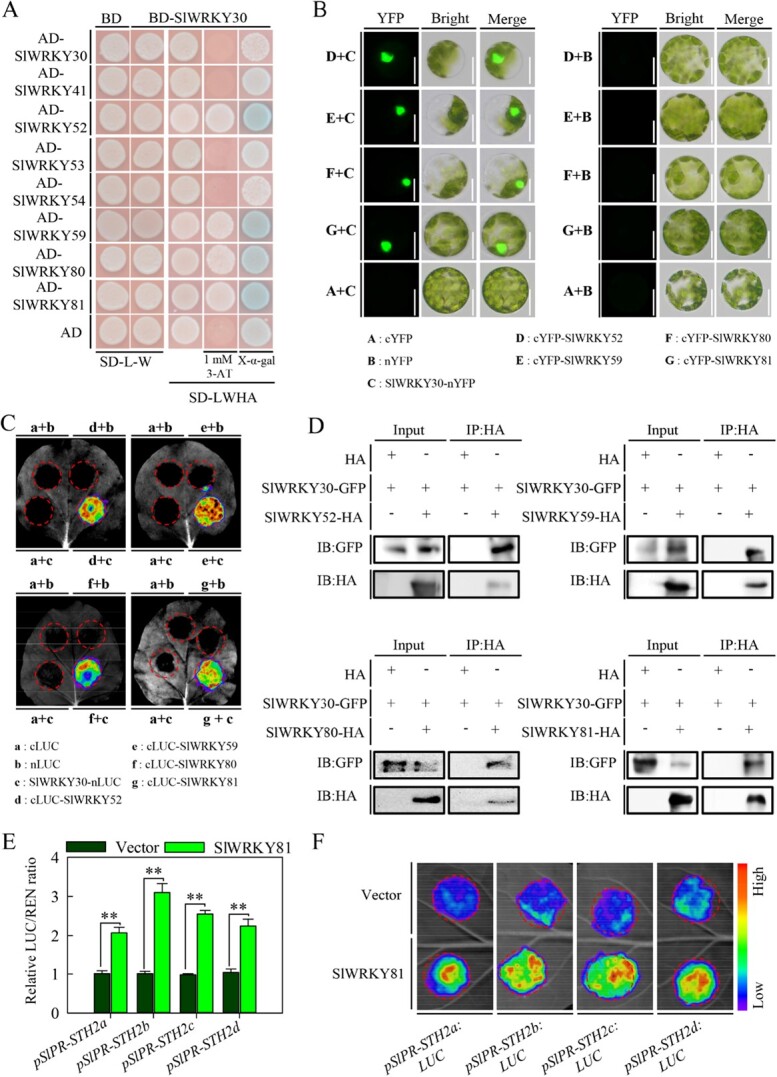
SlWRKY30 interacts with SlWRKY52, SlWRKY59, SlWRKY80, and SlWRKY81. (A) Y2H assay showing that SlWRKY30 interacts with SlWRKY52, 59, 80, and 81. (B) BiFC assay verifying the interactions between SlWRKY30 and SlWRKY52, 59, 80, and 81 in *Arabidopsis* protoplasts. Representative images are shown for protoplast cells at 10 hours after incubation in WI buffer. Scale bar = 20 μm. (C) LCI assay verifying the interactions between SlWRKY30 and SlWRKY52, 59, 80, and 81 in *N. benthamiana* leaves. Representative images of *N. benthamiana* leaves transiently expressing *LUC* reporter gene for each interaction are shown. (D) Co-IP assay verifying the interactions between SlWRKY30 and SlWRKY52, 59, 80, and 81 in *N. benthamiana* leaves. (E) Dual-luciferase assay showing that SlWRKY81 activates expression of the *LUC* reporter gene driven by the *SlPR-STH2a*/*b*/*c*/*d* promoters. Values represent mean ± standard error. (F) Transient expression assay showing that SlWRKY81 transcriptionally activates the *LUC* reporter gene (driven by the *SlPR-STH2a*/*b*/*c*/*d* promoters).

WRKY TFs may be post-translationally modulated by interacting with regulatory proteins, such as themselves or other WRKY TFs, to cooperatively or antagonistically regulate gene transcription [16, 31]. However, few studies have investigated the interacting partners of group III WRKY TFs in plant immunity, especially in non-model plants. To explore the interactions among group III SlWRKY TFs in the tomato response to RSI, we performed a yeast two-hybrid (Y2H) assay between SlWRKY30 and itself and the other seven group III WRKY TFs investigated in this study (SlWRKY41, SlWRKY52, SlWRKY53, SlWRKY54, SlWRKY59, SlWRKY80, and SlWRKY81). SlWRKY30 interacted with SlWRKY52, 59, 80, and 81 in yeast. Notably, SlWRKY30 did not form a homodimer with itself, and did not interact with SlWRKY41, 53, and 54 in yeast (Fig. 6A). To validate these results, we performed a bimolecular fluorescence complementation (BiFC) assay in *Arabidopsis* mesophyll protoplasts. Indeed, SlWRKY30 interacted with SlWRKY52, 59, 80, and 81 (Fig. 6B, Supplementary Data Fig. S7A), but not with itself or SlWRKY41, 53, or 54 (Supplementary Data Fig. S7B). Consistent with this, we observed protein–protein interactions between SlWRKY30 and SlWRKY52, 59, 80, and 81 in *N. benthamiana* leaves by a split luciferase complementation imaging (LCI) assay (Fig. 6C), but did not observe interactions between SlWRKY30 and itself, SlWRKY41, SlWRKY53, or SlWRKY54 (Supplementary Data Fig. S7C). To further confirm the interaction of SlWRKY30 and SlWRKY52, 59, 80, and 81, we performed co-immunoprecipitation (Co-IP) assays using GFP-fused SlWRKY30 and HA-fused SlWRKY52, 59, 80, and 81. Our data showed that SlWRKY30 interacted with SlWRKY52, 59, 80, and 81 in *N. benthamiana* leaves (Fig. 6D). Collectively, these data demonstrate that SlWRKY30 interacts with SlWRKY52, 59, 80, and 81. As all of their corresponding genes were upregulated by RSI in tomato, we speculated that these interactions may modulate SlWRKY30 function during the tomato response to RSI.

### SlWRKY81 positively regulates tomato immunity to *R. solanacearum* infection by regulating *SlPR­STH2a*/*b*/*c*/*d*

To test if SlWRKY30 is modulated by SlWRKY52, 59, 80, and 81 during the response to RSI, we investigated the SlWRKY30–SlWRKY81 interaction, because *SlWRKY81* was most strongly induced in tomato plants after 6 hpi with *R. solanacearum* (Fig. 1). First, to determine the function of SlWRKY81 in tomato immunity to RSI, we generated *SlWRKY81-*silenced tomato plants by VIGS using the *TRV:Slwrky81* construct, and tested the silencing efficiency by RT–qPCR. *SlWRKY81* was downregulated in *TRV:Slwrky81* plants compared with plants transformed with the empty vector control (*TRV:00*) (Supplementary Data Fig. S8A). *SlWRKY81* silencing enhanced tomato susceptibility to RSI compared with the empty vector control plants (Supplementary Data Fig. S8B), exemplified by an increased disease index and *R. solanacearum* growth rate (Supplementary Data Fig. S8C and D). These results indicate that SlWRKY81 positively regulates tomato immunity to RSI, and that SlWRKY30 and SlWRKY81 might synergistically regulate the expression of *SlPR-STH2a*/*b*/*c*/*d*. To validate this, we performed dual-luciferase assays with *SlWRKY81* in *N. benthamiana* leaves. Similar to the results with *SlWRKY30*, transient overexpression of *SlWRKY81* driven by the *CaMV35S* promoter significantly promoted the expression of *LUC* driven by the *SlPR­STH2a*/*b*/*c*/*d* promoters (Fig. 6E and 6F), indicating that SlWRKY81 positively regulates tomato immunity to RSI by cooperating with SlWRKY30 to regulate *SlPR­STH2a*/*b*/*c*/*d* expression.

### SlWRKY30 and SlWRKY81 directly and synergistically regulate *SlPR­STH2a*/*b*/*c*/*d*

**Figure 7:**
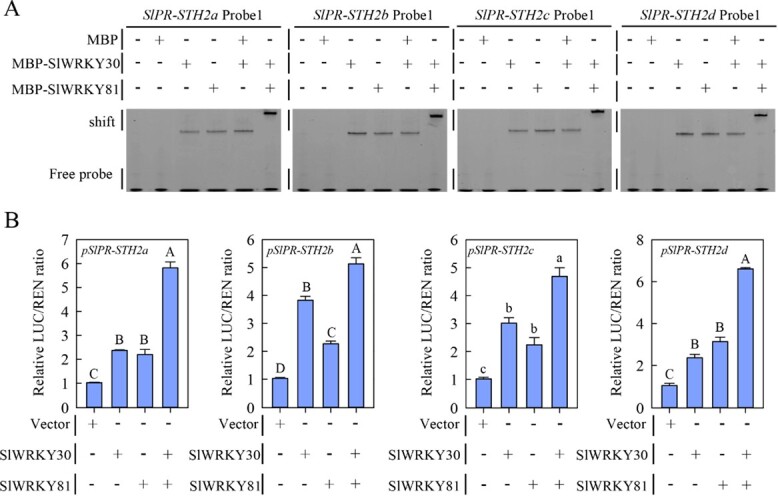
SlWRKY30 and SlWRKY81 synergistically activate *SlPR-STH2a*, *SlPR­STH2b*, *SlPR­STH2c*, and *SlPR-STH2d* expression. (A) EMSA showing that addition of the MBP–SlWRKY81 recombinant protein affected the mobility of MBP–SlWRKY30-bound DNA probe 1 (generated from the *SlPR­STH2a*/*b*/*c*/*d* promoters). (B) Transient expression assay in *N. benthamiana* leaves showing that SlWRKY81 significantly enhanced the transcriptional activation activity of SlWRKY30 on *pSlPR*­*STH2a:LUC*, *pSlPR­STH2b:LUC*, *pSlPR-STH2c:LUC*, and *pSlPR-STH2d:LUC*. Values represent mean ± standard error.

Since SlWRKY30 and SlWRKY81 interact, and both positively regulate tomato immunity to RSI by regulating *SlPR-STH2a*/*b*/*c*/*d* expression, and because *SlPR­STH2a*/*b*/*c*/*d* are directly targeted by SlWRKY30, we speculated that SlWRKY30 and SlWRKY81 synergistically and directly regulate *SlPR-STH2a*/*b*/*c*/*d* expression. To test this, we performed an EMSA with the MBP–SlWRKY81 fusion protein and *SlPR­STH2a*/*b*/*c*/*d* promoter DNA probes. MBP–SlWRKY81, but not MBP alone, directly bound to DNA probes 1 and 2, were repressed by the competitors (Supplementary Data Fig. S9A and B), indicating that SlWRKY81 directly regulates *SlPR­STH2a*/*b*/*c*/*d* expression. To examine if the SlWRKY30–SlWRKY81 interaction affects SlWRKY30 or SlWRKY81 targeting to *SlPR-STH2a*/*b*/*c*/*d*, we performed another EMSA. Both MBP–SlWRKY30 and MBP–SlWRKY81 bound to DNA probe 1, and the mobility of MBP–SlWRKY30-bound DNA probe 1 was altered by addition of MBP*–*SlWRKY81, while addition of MBP alone did not alter mobility (Fig. 7A), indicating that SlWRKY30 interacts with SlWRKY81 and they synergistically bind the *SlPR*­*STH2a*/*b*/*c*/*d* promoters. In addition, transiently expressed *SlWRKY30* or *SlWRKY81* alone activated the expression of *LUC* driven by the *SlPR-STH2a*/*b*/*c*/*d* promoters in *N. benthamiana* leaves (Fig. 7B). However, co-expression of *SlWRKY30* and *SlWRKY81* significantly increased *LUC* expression compared with *SlWRKY30* or *SlWRKY81* alone, indicating that SlWRKY81 promotes SlWRKY30­mediated transactivation of the *SlPR-STH2a*/*b*/*c*/*d* promoters. Collectively, these results demonstrate that SlWRKY30 interacts with SlWRKY81 and that they directly and synergistically activate the expression of *SlPR-STH2a*/*b*/*c*/*d*.

## Discussion

Bacterial wilt, caused by *R. solanacearum*, is one of the most devastating diseases in tomato. WRKY TFs are implicated in plant immunity to various pathogens, but their functions in immunity to *R. solanacearum* in tomato remain poorly understood. Here we report that several group III SlWRKY TFs participate in tomato immunity to RSI; in particular, SlWRKY30 positively regulates immunity, and its function is modulated by other group III SlWRKY TFs through protein–protein interactions. This article provides novel insight into the molecular mechanisms whereby group III SlWRKY TFs regulates immunity, as well as candidate genes for improving tomato resistance to RSI.

### SlWRKY30 and several other group III SlWRKY transcription factors participate in tomato immunity to *R. solanacearum* infection

Genes that are transcriptionally modified upon pathogen attack are often implicated in plant immunity, which is the rationale for many immunity-related transcriptome analyses. Moreover, many group III SlWRKY TFs participate in plant immunity to various pathogens [10, 14]. Therefore, we explored the transcriptional changes of group III *SlWRKY* TF genes using RT–qPCR upon RSI in tomato, as these TFs might participate in tomato immunity to RSI. Five group III *SlWRKY* genes, particularly *SlWRKY30* and *SlWRKY81*, were significantly induced by RSI (Fig. 1), and SlWRKY30 and SlWRKY81 increased tomato immunity to RSI (Fig. 3A–C, Supplementary Data Figs S5 and S8), indicating that several group III SlWRKY TFs positively regulate tomato resistance to bacterial wilt. In addition, phylogenetic analysis revealed that the homolog of SlWRKY30 in pepper is CaWRKY41 (Fig. 2B, Supplementary Data Fig. S3B), which confers *R. solanacearum* resistance in pepper. Considering that tomato and pepper are attacked by similar pathogens, such as *R. solanacearum*, the resistance loci containing *SlWRKY30* and *CaWRKY41* may have been selected during tomato and pepper domestication, respectively. The induction of *SlWRKY30* in leaf veins and the root stele during RSI further supports its role as a positive regulator of tomato immunity (Fig. 3F, Supplementary Data Fig. S4C), since *R. solanacearum* exclusively invades roots [19, 20] and then spreads through the vasculature [27]. Furthermore, the positive role of SlWRKY30 in tomato immunity might be due to regulation of the H_2_O_2_ accumulation and hypersensitive response (HR) cell death (Fig. 3D and E), as H_2_O_2_ promotes HR cell death [32]. HR cell death frequently accompanies ETI [2, 33], and sometimes also PTI [34]; therefore, SlWRKY30 may function in ETI and possibly also PTI. In addition, *SlWRKY30* and several other group III *SlWRKY* genes were upregulated by exogenous application of SA (Supplementary Data Fig. S1A). SA signaling is involved in the RSI response in pepper [26], and HR cell death is also promoted by SA signaling [35], indicating that SlWRKY30 and other group III SlWRKY TFs might function in an SA-dependent manner.

### SlWRKY30 functions in tomato immunity to *R. solanacearum* infection by directly targeting and regulating *SlPR-STH2* genes

The RNA-seq data of *SlWRKY30*-overexpressing tomato lines indicated that SlWRKY30 functions by upregulating multiple genes involved in a broad range of processes, such as ‘defense response’, ‘chitin binding’, and ‘chitin catabolic process’, and also by downregulating processes such as ‘chloroplast thylakoid membrane’, ‘photosystem II’, and ‘chlorophyll binding’ (Fig. 4A and B), indicating that *SlWRKY30* overexpression activated immunity-related genes and repressed genes related to growth and other biological processes, to ensure mobilization of resources for the immune response. Defense-response genes were enriched in the GO analysis of the *SlWRKY30*-overexpression line, including several *SlPR-STH2* genes (*SlPR-STH2a*, *b*, *c*, and *d*) that exist as a gene cluster (Fig. 4C, Supplementary Data Fig. S6A). Some *SlPR­STH2* orthologs in pepper are activated by RSI and positively regulate pepper immunity [26]. These *SlPR-STH2* genes were also directly activated by SlWRKY81, which also positively regulated tomato immunity (Fig. 6D and E, Supplementary Data Figs S8 and S9), indicating that these *PR* genes have important roles in tomato resistance to bacterial wilt. Furthermore, genes in the abscisic acid (ABA) signaling pathway were also activated by SlWRKY30. Studies on the role of ABA in plant immunity have yielded conflicting results. For example, ABA signaling positively regulates *Arabidopsis* resistance to bacterial wilt [36] but negatively regulates pepper immunity to RSI under room temperature. Therefore, the biological relevance of SlWRKY30­mediated upregulation of ABA signaling genes in tomato during the response to RSI needs further investigation.

### SlWRKY30 is post-translationally modified by other group III SlWRKY transcription factors

We found that eight group III SlWRKY TFs were transcriptionally modulated by RSI in tomato. Among them, SlWRKY30, 52, 59, 80, and 81 were upregulated by RSI and by exogenous application of SA, which is implicated in plant immunity to hemibiotrophic pathogens such as *R. solanacearum* [26]. Y2H, BiFC, LCI, and Co-IP analyses revealed that SlWRKY30 interacts with SlWRKY81 (Fig. 6), which also positively regulated tomato immunity to RSI (Supplementary Data Fig. S8), SlWRKY52, 59, and 80, indicating that these TFs and their interaction are involved in tomato immunity to RSI. Protein–protein interactions between group III WRKY TFs are not limited to plant immunity, since *Arabidopsis* AtWRKY30 interacts with AtWRKY53, 54, and 70, which negatively regulate leaf senescence in *Arabidopsis* [37]. Moreover, interactions among group IIa WRKY members AtWRKY18, CaWRKY40, and AtWRKY60 are involved in basal defense [38], indicating that WRKY interactions allow for plasticity in their regulation of different biological processes. We revealed that the promoters of *SlPR­STH2a*/*b*/*c*/*d* are targeted and regulated by SlWRKY30 and SlWRKY81 via two closely spaced W-boxes (Fig. 7). The interaction between SlWRKY30 and SlWRKY81 may further promote the transcriptional expression of their shared target genes over that of either TF alone, as demonstrated in our dual-luciferase assay, which is distinct from the interactions between CaWRKY17 and CaWRKY40 and between CaWRKY27b and CaWRKY40, in which both CaWRKY17 and CaWRKY27b function by physically interacting with CaWRKY40 to promote CaWRKY40 binding to and activation of its immunity-related target genes [16, 39]. Although SlWRKY52, 59, and 80 also interacted with SlWRKY30, their precise roles in the tomato immune response to *R. solanacearum* need further study. *SlPR­STH2a*/*b*/*c*/*d* share ~70% identity in their deduced amino acid sequences, and form a SlPR­STH2 tandem duplication (Supplementary Data Fig. S6A). In soybean (*Glycine max*), the *Rps11* locus, which harbors a cluster of large *NLR* genes from a single origin and results in promoter fusion and leucine-rich repeat (LRR) expansion, confers broad­spectrum resistance to *Phytophthora sojae* [40]. However, whether the tandem duplication of the *SlPR-STH2* genes is responsible for the increased tomato resistance to *R. solanacearum* needs more study. Moreover, it will be interesting to elucidate the molecular mechanism by which the SlWRKY30–SlWRKY81 module promotes the SA­mediated signaling pathway.

### Conclusions

**Figure 8:**
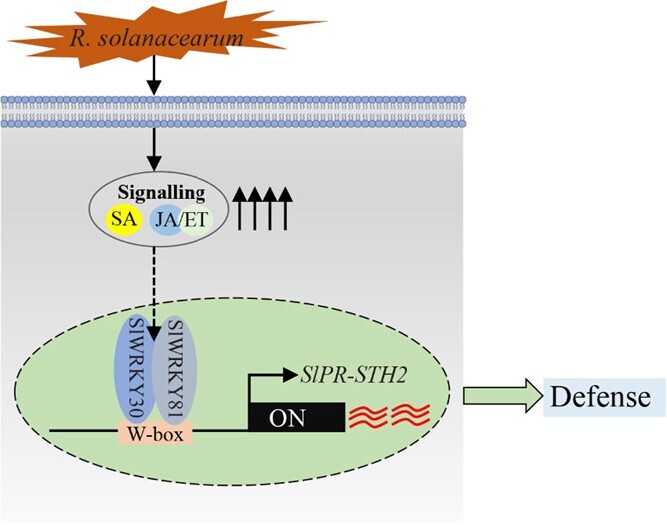
Proposed working model of the SlWRKY30–SlWRKY81 module in regulating tomato resistance to RSI. *R. solanacearum* and phytohormones, such as SA and JA/ACC, induce *SlWRKY30* and *SlWRKY81.* Then, SlWRKY30 interacts with SlWRKY81 to directly and synergistically activate the expression of *SlPR-STH2a*/*b*/*c*/*d*, increasing tomato resistance to RSI.

Discovery of genes that confer resistance to RSI is crucial to preventing bacterial wilt outbreaks in tomato production. We identified two group III SlWRKY TFs, SlWRKY30 and SlWRKY81, that were upregulated by RSI and positively regulated tomato immunity by directly targeting and regulating *SlPR-STH2a*/*b*/*c*/*d*. The function of SlWRKY30 might be modulated via protein–protein interactions with SlWRKY52, 59, 80, and 81. Based on these results, we propose a model of the mechanism by which SlWRKY30 regulates immunity to RSI (Fig. 8).

## Materials and methods

### Plant material and growth conditions

The tomato *S. lycopersicum* L. cv. Micro-Tom was used as the WT genotype for all tomato lines generated in the experiments. The recombinant *SlWRKY30-OE* and *pSlWRKY30*-*GUS* plasmids were individually transformed into *Agrobacterium tumefaciens* (strain GV3101), and the WT tomato plants were transformed through *A. tumefaciens*-mediated infection. Plants were grown on media to select for hygromycin B resistance, and nine *SlWRKY30-OE* and eight *pSlWRKY30:GUS* independent transgenic lines were generated and screened using PCR/RT–qPCR analysis and GUS assay, respectively. Then, the *SlWRKY30­OE6* (~14­fold) and *SlWRKY30-OE8* (~12-fold) *T*_2_ or *T*_3_ seeds were used for the experiments. The tomato seeds were germinated at 25 ± 2°C in darkness for 48 hours on moistened sterile filter paper, then sown in a steam-sterilized soil mixture, which included peat moss and vermiculite (2:1 by volume). Subsequently, tomato seedlings were transferred to a temperature (25 ± 2°C)-controlled growth room under a light intensity of ~100 μmol photons m^−2^ s^−1^ and a 16-hour light/8-hour dark cycle.

### Molecular cloning and plasmid construction

To generate the *SlWRKY30-OE* construct, the coding sequence (CDS) of full-length *SlWRKY30* was PCR-amplified from tomato cDNA, cloned into the *pDONR207* vector, then recombined with the binary vector *pGWB2* [41]. To generate the *pSlWRKY30:GUS* construct, the 2.0-kb *SlWRKY30* promoter was PCR-amplified, cloned into *pDONR207*, and recombined with the binary vector *pMDC163* [42]. To generate the DNA constructs for transcriptional activity and subcellular localization analyses, the CDSs of *SlWRKY30*, *41*, *52*, *53*, *54*, *59*, *80*, and *81* were PCR-amplified from tomato cDNA and cloned into vectors *pGBKT7* and *35S:GFP* (*35S-CDS-NOS Terminator*) [43]. PCR primers used in the DNA constructs are listed in Supplementary Data Table S1.

### Pathogens and inoculation procedures

For the pathogenicity test, 5-week-old *SlWRKY30-OE6*, *SlWRKY30-OE8*, and WT tomato plants were infected with a suspension of *R. solanacearum* (strain FJ190401). The mean number of colony forming units (CFU) of five leaves from various tomato genotypes was recorded at 0, 24, and 48 hpi. Then, the disease index (from 0 to 5) of the *R. solanacearum*­infected tomato plants was scored daily as described previously [42].

### Subcellular localization and transcriptional activity analyses

For subcellular localization, the *35S-SlWRKY-GFP* plasmids of the different group III WRKYs were isolated and purified as described previously [44]. Subcellular localization of SlWRKY–GFP fusion proteins was conducted in *A. thaliana* mesophyll protoplasts (200 μl) transfected with 20 μg plasmid DNA and incubated in WI buffer [45] for 10 hours. Then GFP fluorescence was observed using a fluorescence microscope (Carl Zeiss, Germany).

For transcriptional activity analysis, the *BD*-*SlWRKY* constructs were transformed into AH109 yeast cells and grown on SD/−Trp medium at 30°C for 48–60 hours, and then the positive transformants were transferred to SD/−Trp medium containing X-*α*-Gal (5­bromo-4-chloro-3-indolyl-α-d-galactopyranoside) as substrate for blue color development.

### Virus-induced gene silencing

For VIGS assays [46], the *SlWRKY30* and *SlWRKY81* gene fragments were identified by BLAST searching in the tomato genome sequences. These fragments were cloned into a tobacco rattle virus VIGS vector (*pYL-279*) using the Gateway system (Invitrogen). Vectors *TRV:Slwrky30*, *TRV:Slwrky81*, and *TRV:00* (empty vector) were transformed into *A. tumefaciens*, respectively; subsequently, they were mixed with *A. tumefaciens* harboring *pYL*­*192* at a 1:1 ratio, their concentration was adjusted to OD_600_ = 0.5, and then they were injected into fully expanded tomato seedling leaves.

### Histochemical staining

For 3,3′-diaminobenzidine (DAB) and trypan blue staining, 4-week-old tomato plants were inoculated with a suspension of *R. solanacearum*. At 48 hpi, leaves were harvested and stained with DAB or trypan blue solution, as described previously [42].

For GUS staining, leaves and root tissues were immersed for 12 hours in GUS staining solution, as described previously [41], at 37°C. Then, the leaves were destained several times using 75% (v/v) ethanol. The samples were photographed with a stereo microscope (Leica, Germany).

### Transient expression dual-luciferase assay

To generate the *pSlPR-STH2ap:LUC*, *pSlPR­STH2bp:LUC*, *pSlPR­STH2cp:LUC*, and *pSlPR-STH2dp:LUC* constructs, the *SlPR­STH2a*/*b*/*c*/*d* promoter fragments were cloned into *pGreenII*­*0800*­*LUC* vector [47], which was digested with HindIII*-* and BamHI. To generate the *p62*­*SlWRKY30* and *p62*­*SlWRKY81* constructs, the *SlWRKY30* and *SlWRKY81* CDSs were inserted into *pGreenII 62*­*SK* vector [47], which was digested with BamHI and HindIII. The transient expression dual-luciferase assay was performed in *N*. *benthamiana* leaves as described previously [47]. To determine the LUC/REN ratio, the values of *LUC* reporter expression were normalized to the values of *REN* luciferase expression, which was driven by the *35S* promoter.

### Electrophoretic mobility shift assay

According to the EMSA protocol, the *SlWRKY30* and *SlWRKY81* CDSs were amplified by PCR using primers MBP-SlWRKY30-F/R and MBP-SlWRKY81-F/R and cloned into *pMAL*­*c4X* (NEB, http://www.neb-china.com/). Then, MBP–SlWRKY30 and MBP–SlWRKY81 fusion proteins were induced at 16°C for 16–20 hours by 0.4 mM β­d­1­thiogalactopyranoside (IPTG), and purified with Amylose Resin (NEB). The concentration of MBP–SlWRKY30 and MBP–SlWRKY81 was determined by bovine serum albumin (BSA) quantitative analysis. Then, 2 μg of purified MBP–SlWRKY30 or MBP–SlWRKY81 was incubated with Cy5-labeled probes in 20 μl reaction mixtures at 25°C for 30 minutes and then separated in Tris–glycine buffer with 12% native polyacrylamide gels. Unlabeled WT probes were used as competitors. The gel image was visualized through a LI-COR Odyssey Infrared Imaging System (LI-COR, USA) to detect the Cy5-labeled probes. The sequences of Cy5-labeled probes are listed in Supplementary Data Table S1.

### Yeast two-hybrid assay, bimolecular fluorescence complementation, and luciferase complementation imaging

For the Y2H assay, the *SlWRKY30* CDS was cloned into the *pGBKT7* vector, and the *SlWRKY30*, *41*, *52*, *53*, *54*, *59*, *80*, and *81* CDSs were cloned into the *pGADT7* vector. For the BiFC assay, the *SlWRKY30* CDS was inserted into the *35S:nYFP* vector, and the *SlWRKY30*, *41*, *52*, *53*, 5*4*, *59*, *80*, and *81* CDSs were inserted into the *35S:cYFP* vector [43]. For LCI assays, the *SlWRKY30* CDS was cloned into the *pCAMBIA-nLUC* vector [48], which was digested using KpnI- and SalI. The *SlWRKY30*, *41*, *52*, *53*, 5*4*, *59*, *80*, and *81* CDSs were cloned into the *pCAMBIA-cLUC* vector [48], which was digested using KpnI- and SalI. Subsequently, the Y2H, BiFC, and LCI assays were carried out as described previously [44]*.*

### Co-immunoprecipitation assay

For Co-IP assays, the *35S:SlWRKY30-GFP*, *35S:SlWRKY52-HA*, *35S:SlWRKY59-HA*, *35S:SlWRKY80-HA*, *35S:SlWRKY81-HA*, and 3*5S:HA* (as a negative control) constructs were transformed into *A. tumefaciens* (GV3101). *N. benthamiana* leaves were infiltrated with *A. tumefaciens* suspensions containing the respective constructs, followed by incubation at 25°C for 36–48 hours (16-hours light/8-hours dark cycle). Subsequently, total proteins were extracted from the samples using protein extraction buffer [50 mM Tris–HCl (pH 7.5), 150 mM MaCl, 10 mM MgCl_2_, 0.1% (v/v) Tween 20, 1 mM PMSF, and 1 × protease inhibitor cocktail (Roche)] and incubated with magnetic GPF-trap beads (Chromotek, Germany) at 4°C for 2 hours. After washing three times with Co-IP buffer [50 mM Tris–HCl (pH 7.5), 150 mM NaCl, 1 mM EDTA, 1 mM dithiothreitol, 10% (v/v) glycerol, 0.25% IGEPAL CA-630, and 1 mM PMSF], the samples were separated through 12% SDS–PAGE. For immunoblot analysis, anti-GFP (M20004, Abmart, Shanghai, China, 1/5000) and anti-HA (M20003, Abmart, Shanghai, China, 1/3000) antibodies were used to detect the levels of SlWRKY30 or SlWRKY52/59/80/81proteins, respectively.

### RNA sequencing

Four-week-old WT and *SlWRKY30-OE* (OE6) plants were infected using *R. solanacearum* suspensions. At 24 hpi, samples were harvested from three biological replicates, and total RNA was extracted using the TRIzol kit (Invitrogen) following the user manual. RNA quality was tested by a NanoDrop spectrophotometer, and verified by an Agilent 2100 Bioanalyzer. Then, cDNA libraries were constructed for sequencing by Biomarker Technologies (China). The DEGs were identified and filtered based on the following criteria: fold change ≥2 and false discovery rate <.01. The raw sequencing reads (RNA-seq data) have been deposited in the China National GeneBank DataBase (CNGBdb, https://db.cngb.org/cnsa/) under accession number CNP0003958. A summary of the sequencing data is shown in Supplementary Data Table S2. The top 20 enriched functions of the DEGs in the RNA-seq data sets were selected by GO term enrichment analysis. A summary of the analysis is shown in Supplementary Data Tables S3 and S4.

### Gene expression analysis

Gene expression data were analyzed using RT–qPCR, as described previously [42]. In brief, total RNA was extracted and then used as a template to synthesize cDNA using TaKaRa PrimeScript RT-PCR Kit (TaKaRa) following the user manual. Quantitative PCR was conducted as previously described [42]. The 2^-ΔΔCt^ method was used to calculated the relative expression of target genes, and normalized to that of the tomato internal reference gene *SlACTIN2*. For each sample, three independent biological replicates were performed.

### Statistical analysis

Differences were analyzed by Student’s *t* test (two groups) or Tukey’s multiple comparisons test (four groups). The significances of differences were determined using the *P* value. Statistically significant differences are indicated in the figures with asterisks (^*^P < .05 or ^**^P < .01) or different letters (lowercase letters, *P* < .05; uppercase letters, *P* < .01, Tukey’s multiple comparisons test). All experiments were performed and analyzed with at least three biological replicates.

### Accession numbers

Sequence information from this article can be found in the Sol Genomics Network Initiative (https://solgenomics.net/) under the following accession numbers: *SlWRKY30* (Solyc10g009550), *SlWRKY41* (Solyc01g095630), *SlWRKY52* (Solyc03g007380), *SlWRKY53* (Solyc08g008280), *SlWRKY54* (Solyc08g082110), *SlWRKY59* (Solyc05g050330), *SlWRKY80* (*Solyc03g095770*), *SlWRKY81* (Solyc09g015770), *SlPR-STH2a* (Solyc09g090970), *SlPR-STH2b* (Solyc09g090980), *SlPR-STH2c* (Solyc09g090990), *SlPR-STH2d* (Solyc09g091000), and *SlACTIN2* (Solyc11g005330).

## Supplementary Material

Web_Material_uhad050

## Data Availability

All relevant data supporting our findings are available in the manuscript file or from the corresponding author upon request.
