# Correction to: A mutation in the brassinosteroid biosynthesis gene *CpDWF5* disrupts vegetative and reproductive development and the salt stress response in squash (*Cucurbita pepo*)

**DOI:** 10.1093/hr/uhae217

**Published:** 2024-08-10

This is a correction to: Sonsoles Alonso, Gustavo Cebrián, Keshav Gautam, Jessica Iglesias-Moya, Cecilia Martínez, Manuel Jamilena, A mutation in the brassinosteroid biosynthesis gene *CpDWF5* disrupts vegetative and reproductive development and the salt stress response in squash (*Cucurbita pepo*), *Horticulture Research*, Volume 11, Issue 4, April 2024, https://doi.org/10.1093/hr/uhad050

In the originally published version of the manuscript, there was an error affecting Figure 6, panels G and H in the omission of the names of the genes studied by qRT-PCR.

Figure 6 should read:


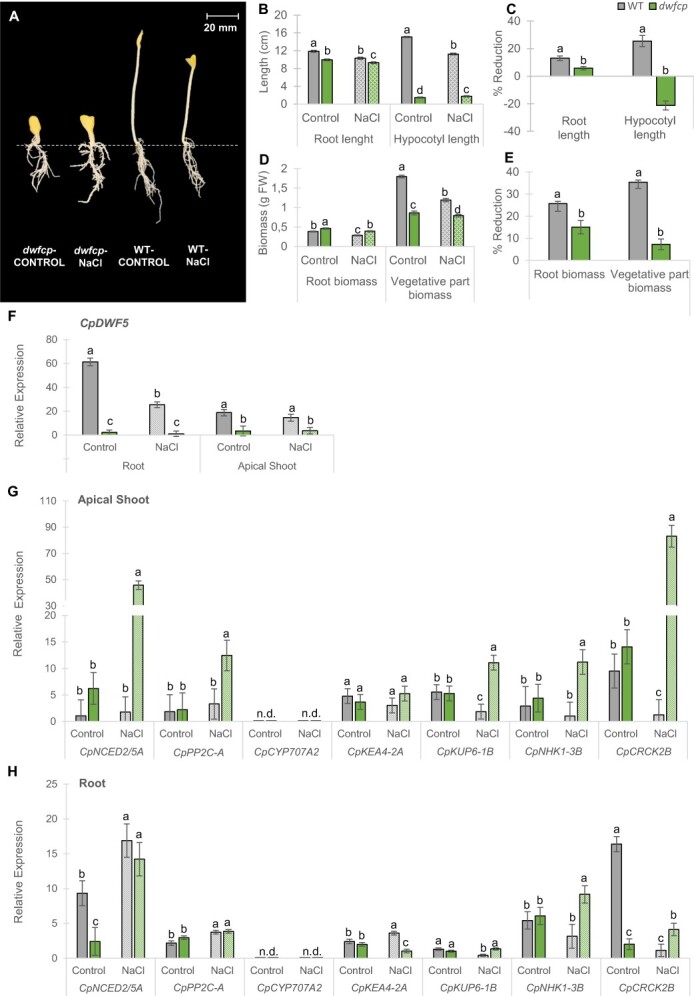


instead of:


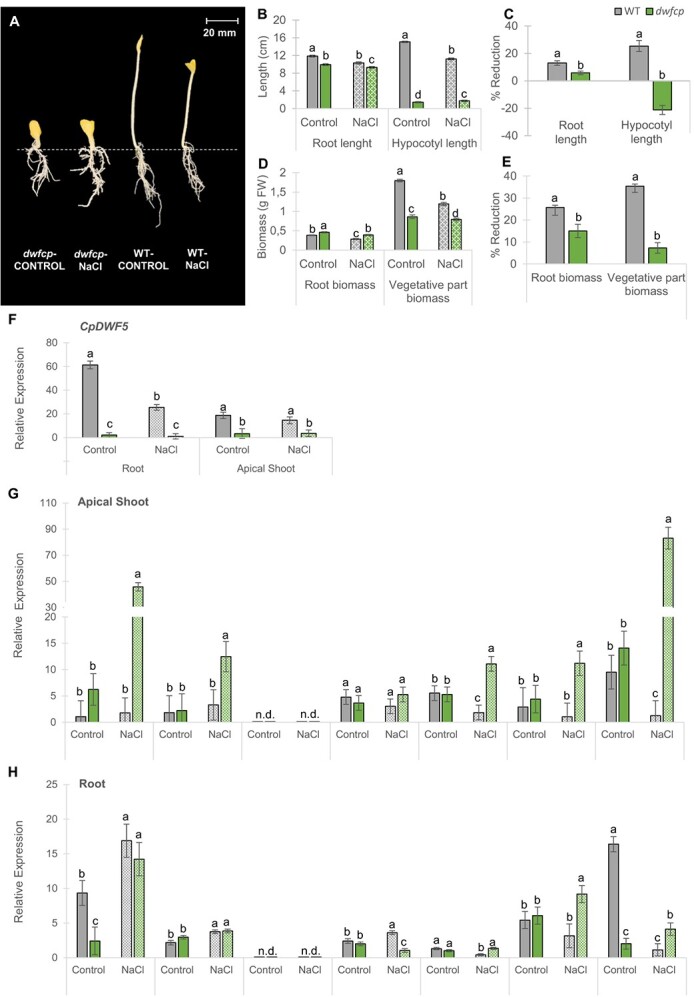


The emendations have been made to the figure in the article.

