# Supplementary material for: SlWRKY30 and SlWRKY81 synergistically modulate tomato immunity to Ralstonia solanacearum by directly regulating SlPR-STH2
Source: Hortic Res. 2023 Mar 15;10(5):uhad050. doi: 10.1093/hr/uhad050 (PMC10189802; doi:10.1093/hr/uhad050)
Supplement: Web_Material_uhad050 [file web_material_uhad050.zip › SlWRKY30 Supplemental Table 1-2-22.docx]

**Supplemental Table1. List of the primers used in this study**

| **Primers used for DNA constructs** | | |
| --- | --- | --- |
| **Primer Name** | **Forward (5′-3′)** | **Reverse(5′-3′)** |
| SlWRKY30-pGWB2 | ggggacaagtttgtacaaaaaagcaggcttcATGGAGAAAGTTAAAGTTTG | ggggaccactttgtacaagaaagctgggtcTTAGGTGAAATATTCAGAAAT |
| SlWRKY30pro-pMDC163 | ggggacaagtttgtacaaaaaagcaggcttcGTGTGAATAGTAGTATCTCA | ggggaccactttgtacaagaaagctgggtcAAATGGGGTGTTTCAAAACA |
| **Primers used for virus-induced gene silencing** | | |
| \| SlWRKY30-pYL-279 \| ggggacaagtttgtacaaaaaagcaggcttcCAGCAATGGAGCAAACAAGT \| ggggaccactttgtacaagaaagctgggtcTGTTGCTAAGCAGCCTTGTG \| \| --- \| --- \| --- \| \| SlWRKY30pro-pMDC163 \| ggggacaagtttgtacaaaaaagcaggcttcGTGTGAATAGTAGTATCTCA \| ggggaccactttgtacaagaaagctgggtcAAATGGGGTGTTTCAAAACA \| | | |
| \| SlWRKY81- pYL-279 \| gggacaagtttgtacaaaaaagcaggcttcGGACTCACACTTGCAAAGC \| gggaccactttgtacaagaaagctgggtcAGGCTTCAAATGTTGCTGGA \| \| --- \| --- \| --- \| | | |
| **Primers used fortranscriptional activity and subcellular localization** | | |
| BD-SlWRKY30 | catggaggccgaattcATGGAGAAAGTTAAAGTTTG | gcaggtcgacggatccGGTGAAATATTCAGAAATGT |
| BD-SlWRKY41 | catggaggccgaattcATGGAGAAAGTTAAAAGTAT | gcaggtcgacggatccAATGAAGAATTCTTCAAT |
| BD-SlWRKY52 | catggaggccgaattcATGGATAATTACGGAGCTGA | gcaggtcgacggatccAGAGAATCTGTGATAATC |
| BD-SlWRKY53 | catggaggccgaattcATGGATTGTGCATCAAACTG | gcaggtcgacggatccTGAGAAAAATTTGGGGTT |
| BD-SlWRKY54 | catggaggccgaattcATGGATTGTGGATTCAATTA | gcaggtcgacggatccTCTGAAAAAATCAGAGA |
| BD-SlWRKY59 | catggaggccgaattcATGGGAGAAAACAGCTTTTG | gcaggtcgacggatccTGAGAATAACTGTAGG |
| BD-SlWRKY80 | catggaggccgaattcATGGAGGATTTTCTTGGTGA | gcaggtcgacggatccACAAAACTCAATGGCCT |
| BD-SlWRKY81 | catggaggccgaattcATGGATAACTCATCGTCTGA | gcaggtcgacggatccCACTTGATCAAAGTTCCA |
| SlWRKY30-GFP | gacaagcttggtacctctagaATGGAGAAAGTTAAAGTTTG | gcccttgctcaccatactagtGGTGAAATATTCAGAAATGT |
| SlWRKY41-GFP | gcccttgctcaccatactagtATGGAGAAAGTTAAAAGTAT | gcccttgctcaccatactagtAATGAAGAATTCTTCAAT |
| SlWRKY52-GFP | gacaagcttggtacctctagaATGGATAATTACGGAGCTGA | gcccttgctcaccatactagtAGAGAATCTGTGATAATC |
| SlWRKY53-GFP | gacaagcttggtacctctagaATGGATTGTGCATCAAACTG | gcccttgctcaccatactagtTGAGAAAAATTTGGGGTT |
| SlWRKY54-GFP | gacaagcttggtacctctagaATGGATTGTGGATTCAATTA | gcccttgctcaccatactagtTCTGAAAAAATCAGAGA |
| SlWRKY59-GFP | gacaagcttggtacctctagaATGGGAGAAAACAGCTTTTG | gcccttgctcaccatactagtTGAGAATAACTGTAGG |
| SlWRKY80-GFP | gacaagcttggtacctctagaATGGAGGATTTTCTTGGTGA | gcccttgctcaccatactagtACAAAACTCAATGGCCT |
| SlWRKY81-GFP | gacaagcttggtacctctagaATGGATAACTCATCGTCTGA | gcccttgctcaccatactagtCACTTGATCAAAGTTCCA |
| **Primers used for LUC/REN ration and EMSA** | | |
| SlWRKY30-62SK | cgctctagaactagtggatccATGGAGAAAGTTAAAGTTTG | gtcgacggtatcgataagcttTTAGGTGAAATATTCAGAAAT |
| SlSTH2a-0800 | gtcgacggtatcgataagcttCACAATAATATATCGAAATTC | cgctctagaactagtggatccTTTATATATTGCATCTTTTATTAG |
| SlSTH2b-0800 | gtcgacggtatcgataagcttCGATTCAATCTACCTTGCTCAAG | cgctctagaactagtggatccTTTAGGGGTGTATTTTGCTCACG |
| SlSTH2c-0800 | gtcgacggtatcgataagcttTAAAGCCATTTGGTAATGTG | cgctctagaactagtggatccATATTATTTTTCGTGATTTG |
| SlSTH2d-0800 | gtcgacggtatcgataagcttATGGAGGGCATTTTTGTTC | cgctctagaactagtggatccATAATGAGATATTGTTTTATG |
| SlWRKY30-MBP | aaggatttcagaattcATGGAGAAAGTTAAAGTTTGGAATAAAG | gcaggtcgactctgacttaGGTGAAATATTCAGAAATGTCGAT |
| SlWRKY81-MBP | aaggatttcagaattcATGGATAACTCATCGTCTGATCTAAATAG | gcaggtcgactctgacttaCACTTGATCAAAGTTCCAAAGACCC |
| SlPR-STH2a-*Cy5*-EMSA1  (WT) | ACTGACGACTGAAACATATTAAAGAAACAGATCCAACGCAGTCAACTGAC | GTCAGTTGACTGCGTTGGATCTGTTTCTTTAATATGTTTCAGTCGTCAGT |
| SlPR-STH2a-*Cy5*-EMSA1  (mut) | ACTaAaGACTGAAACATATTAAAGAAACAGATCCAACGCAGTCAACTaAa | tTtAGTTGACTGCGTTGGATCTGTTTCTTTAATATGTTTCAGTCtTtAGT |
| SlPR-STH2a-*Cy5*-EMSA2  (WT) | ATCGATTGACATCTGTTCTCCCATATTTAATTCGTCATTGAGGTTTGCTT | AAGCAAACCTCAATGACGAATTAAATATGGGAGAACAGATGTCAATCGAT |
| SlPR-STH2a-*Cy5*-EMSA2 (mut) | ATCGATTaAaATCTGTTCTCCCATATTTAATTCGTCATTGAGGTTTGCTT | AAGCAAACCTCAATGACGAATTAAATATGGGAGAACAGATtTtAATCGAT |
| SlPR-STH2b-*Cy5*-EMSA1 (WT) | CTAGTTTCATTCTCAAACTATTGACAGCCCTAAAAACACCCTTTTACT | AGTAAAAGGGTGTTTTTAGGGCTGTCAATAGTTTGAGAATGAAACTAG |
| SlPR-STH2b-*Cy5*-EMSA1 (mut) | CTAGTTTCATTCTCAAACTATTaAaAGCCCTAAAAACACCCTTTTACT | AGTAAAAGGGTGTTTTTAGGGCTtTtAATAGTTTGAGAATGAAACTAG |
| SlPR-STH2b-*Cy5*-EMSA2 (WT) | TTTACATGATATAGTTAGACTTGACATAAAATTTAAGAAA | TTTCTTAAATTTTATGTCAAGTCTAACTATATCATGTAAA |
| SlPR-STH2b-*Cy5*-EMSA2 (mut) | TTTACATGATATAGTTAGACTTaAaATAAAATTTAAGAAA | TTTCTTAAATTTTATtTtAAGTCTAACTATATCATGTAAA |
| SlPR-STH2c-*Cy5*-EMSA1  (WT) | TTTGATAACGTGACCGAGTTACAATTGGCCACGTGTCAAAAA | TTTTTGACACGTGGCCAATTGTAACTCGGTCACGTTATCAAA |
| SlPR-STH2c-*Cy5*-EMSA1  (mut) | TTTGATAACGTaAaCGAGTTACAATTGGCCACGTGTCAAAAA | TTTTTGACACGTGGCCAATTGTAACTCGtTtACGTTATCAAA |
| SlPR-STH2c-*Cy5*-EMSA2  (WT) | GATCGAATTATAATTGGTCACGTCATCATTTTTCAATAAA | TTTATTGAAAAATGATGACGTGACCAATTATAATTCGATC |
| SlPR-STH2c-*Cy5*-EMSA2  (mut) | GATCGAATTATAATTGtTtACtTtATCATTTTTCAATAAA | TTTATTGAAAAATGATaAaGTaAaCAATTATAATTCGATC |
| SlPR-STH2d-*Cy5*-EMSA1  (WT) | TAGTTTCGACTCTAACTATGGATCCTGACACGGTTTAACTTATTC | GAATAAGTTAAACCGTGTCAGGATCCATAGTTAGAGTCGAAACTA |
| SlPR-STH2d-*Cy5*-EMSA1  (mut) | TAGTTTCGACTCTAACTATGGATCCTaAaACGGTTTAACTTATTC | GAATAAGTTAAACCGTtTtAGGATCCATAGTTAGAGTCGAAACTA |
| SlPR-STH2d-*Cy5*-EMSA2  (WT) | CATGTTTAGTTGACTTATATGTTTTCTAAATAGATTTATT | AATAAATCTATTTAGAAAACATATAAGTCAACTAAACATG |
| SlPR-STH2d-*Cy5*-EMSA2  (mut) | CATGTTTAGTTaAaTTATATGTTTTCTAAATAGATTTATT | AATAAATCTATTTAGAAAACATATAAtTtAACTAAACATG |
| **Primers used for Y2H, BiFC, LCI, and Co-IP** | | |
| AD-SlWRKY30 | gccatggaggccagtgaattcATGGAGAAAGTTAAAGTTTGG | cagctcgagctcgatggatccTTAGGTGAAATATTCAGAAA |
| AD-SlWRKY41 | gccatggaggccagtgaattcATGGAGAAAGTTAAAAGTAT | cagctcgagctcgatggatccTTAAATGAAGAATTCTTCAAT |
| AD-SlWRKY52 | gccatggaggccagtgaattcATGGATAATTACGGAGCTGA | cagctcgagctcgatggatccTTAAGAGAATCTGTGATAATC |
| AD-SlWRKY53 | gccatggaggccagtgaattcATGGATTGTGCATCAAACTG | cagctcgagctcgatggatccTCATGAGAAAAATTTGGGGTT |
| AD-SlWRKY54 | gccatggaggccagtgaattcATGGATTGTGGATTCAATTA | cagctcgagctcgatggatccTTATCTGAAAAAATCAGAGA |
| AD-SlWRKY59 | gccatggaggccagtgaattcATGGGAGAAAACAGCTTTTG | cagctcgagctcgatggatccTTATGAGAATAACTGTAGG |
| AD-SlWRKY80 | gccatggaggccagtgaattcATGGAGGATTTTCTTGGTGA | cagctcgagctcgatggatccTCAACAAAACTCAATGGCCT |
| AD-SlWRKY81 | gccatggaggccagtgaattcATGGATAACTCATCGTCTGA | cagctcgagctcgatggatccCTACACTTGATCAAAGTTCCA |
| SlWRKY30-nYFP | gacaagcttggtacctctagaATGGAGAAAGTTAAAGTTTGG | caacttttgctccatactagtGGTGAAATATTCAGAAATGTC |
| AtSPX1-nYFP | gacaagcttggtacctctagaATGAAGTTTGGTAAGAGTCT | caacttttgctccatactagtTTTGGCTTCTTGCTCCAACAATGG |
| cYFP-SlWRKY30 | gttccagattacgctggatccATGGAGAAAGTTAAAGTTTGG | gagctcgagttataaactagtTTAGGTGAAATATTCAGAAA |
| cYFP-SlWRKY41 | gttccagattacgctggatccATGGAGAAAGTTAAAAGTAT | gagctcgagttataaactagTTAAATGAAGAATTCTTCAAT |
| cYFP-SlWRKY52 | gttccagattacgctggatccATGGATAATTACGGAGCTGA | gagctcgagttataaactagTTAAGAGAATCTGTGATAATC |
| cYFP-SlWRKY53 | gttccagattacgctggatccATGGATTGTGCATCAAACTG | gagctcgagttataaactagTCATGAGAAAAATTTGGGGTT |
| cYFP-SlWRKY54 | gttccagattacgctggatccATGGATTGTGGATTCAATTA | gagctcgagttataaactagTTATCTGAAAAAATCAGAGA |
| cYFP-SlWRKY59 | gttccagattacgctggatccATGGGAGAAAACAGCTTTTG | gagctcgagttataaactagTTATGAGAATAACTGTAGG |
| cYFP-SlWRKY80 | gttccagattacgctggatccATGGAGGATTTTCTTGGTGA | gagctcgagttataaactagTCAACAAAACTCAATGGCCT |
| cYFP-SlWRKY81 | gttccagattacgctggatccATGGATAACTCATCGTCTGA | gagctcgagttataaactagCTACACTTGATCAAAGTTCCA |
| cYFP-AtPHR1 | gttccagattacgctggatccATGGAGGCTCGTCCAGTTCA | gagctcgagttataaactagTCAATTATCGATTTTGGGAC |
| SlWRKY30-nLUC | gagaacacgggggacgagctcggtaccATGGAGAAAGTTAAAGTTTG | gacgcgtacgagatctggtcgacGGTGAAATATTCAGAAATGT |
| cLUC-SlWRKY30 | ctcgtacgcgtcccggggcggtaccATGGAGAAAGTTAAAGTTTG | cgaaagctctgcaggtcgacGGTGAAATATTCAGAAATGT |
| cLUC-SlWRKY41 | ctcgtacgcgtcccggggcggtaccATGGAGAAAGTTAAAAGTAT | cgaaagctctgcaggtcgacTTAAATGAAGAATTCTTCAAT |
| cLUC-SlWRKY52 | ctcgtacgcgtcccggggcggtaccATGGATAATTACGGAGCTGA | cgaaagctctgcaggtcgacTTAAGAGAATCTGTGATAATC |
| cLUC-SlWRKY53 | ctcgtacgcgtcccggggcggtaccATGGATTGTGCATCAAACTG | cgaaagctctgcaggtcgacTCATGAGAAAAATTTGGGGTT |
| cLUC-SlWRKY54 | ctcgtacgcgtcccggggcggtaccATGGATTGTGGATTCAATTA | cgaaagctctgcaggtcgacTTATCTGAAAAAATCAGAGA |
| cLUC-SlWRKY59 | ctcgtacgcgtcccggggcggtaccATGGGAGAAAACAGCTTTTG | cgaaagctctgcaggtcgacTTATGAGAATAACTGTAGG |
| cLUC-SlWRKY80 | ctcgtacgcgtcccggggcggtaccATGGAGGATTTTCTTGGTGA | cgaaagctctgcaggtcgacTCAACAAAACTCAATGGCCT |
| cLUC-SlWRKY81 | ctcgtacgcgtcccggggcggtaccATGGATAACTCATCGTCTGA | cgaaagctctgcaggtcgacCTACACTTGATCAAAGTTCCA |
| SlWRKY52-HA-207 | ggggacaagtttgtacaaaaaagcaggcttcATGGATAATTACGGAGCTGA | ggggaccactttgtacaagaaagctgggtcAGAGAATCTGTGATAATC |
| SlWRKY59-HA-207 | ggggacaagtttgtacaaaaaagcaggcttcATGGGAGAAAACAGCTTTTG | ggggaccactttgtacaagaaagctgggtcTGAGAATAACTGTAGG |
| SlWRKY80-HA-207 | ggggacaagtttgtacaaaaaagcaggcttcATGGAGGATTTTCTTGGTGA | ggggaccactttgtacaagaaagctgggtcACAAAACTCAATGGCCT |
| SlWRKY81-HA-207 | ggggacaagtttgtacaaaaaagcaggcttcATGGATAACTCATCGTCTGA | ggggaccactttgtacaagaaagctgggtcCACTTGATCAAAGTTCCA |
| **Primers used for RT-qPCR** | | |
| *SlWRKY30*  (Solyc10g009550) | TTTGATCCTCTGGTTCACC | GCTCCATTGCTGCATTTTCT |
| *SlWRKY41*  (Solyc01g095630) | AACCAAAAACGCCAGAAATG | TTTGGGGACGCCAAGTATAA |
| *SlWRKY52*  (Solyc03g007380) | CAGCACCACCACCTTTACCT | GTTGGACGACGATGAAGGAT |
| *SlWRKY53*  (Solyc08g008280) | AGCAGATGAAATGGTTACGG | GGGGTTACCAAATGGGAAGT |
| *SlWRKY54*  (Solyc08g082110) | GCAACAAAGCAAGTGCAGAG | TGTAAGTTCGCGAGCATTTG |
| *SlWRKY59*  (Solyc05g050330) | AGCTAACCAAGGGGAAGGAA | TTGATGAAGAATGGTGCTGGA |
| *SlWRKY80*  (Solyc03g095770) | AGTTTAACCCAGGGCCAGAT | TCTCCATGCACAACCATCAT |
| *SlWRKY81*  (Solyc09g015770) | GGACATCACACTTGCAAAGC | AGGCTTCAAATGTTGCTGGA |
| *SlPR-STH2a* (Solyc09g090970) | ACATCATGGGTGTCACTAGCT | TACTTCCATCTCCCTCAGCC |
| *SlPR-STH2b*  (Solyc09g090980) | GCATACCTTCTCGCGAATCC | CAACTTCTACGTCTCTCATTCGA |
| *SlPR-STH2c*  (Solyc09g090990) | TGTTGAGGGAGATGGTGGTG | TCCATCTCCAGCAGCTTCAA |
| *SlPR-STH2d*  (Solyc09g091000) | GACTGAGGGAGATGGAAGCA | CAACCTCCATTTCCAGCAGG |
| *SlACTIN2*  (Solyc11g005330) | GTCCTCTTCCAGCCATCCAT | ACCACTGAGCACAATGTTACCG |
