# Supplementary material for: SlWRKY30 and SlWRKY81 synergistically modulate tomato immunity to Ralstonia solanacearum by directly regulating SlPR-STH2
Source: Hortic Res. 2023 Mar 15;10(5):uhad050. doi: 10.1093/hr/uhad050 (PMC10189802; doi:10.1093/hr/uhad050)
Supplement: Web_Material_uhad050 [file web_material_uhad050.zip › SlWRKY30 Supplemental Figure 1 to 9-2-22.docx]

**Supplemental Figures and Figure Legends**


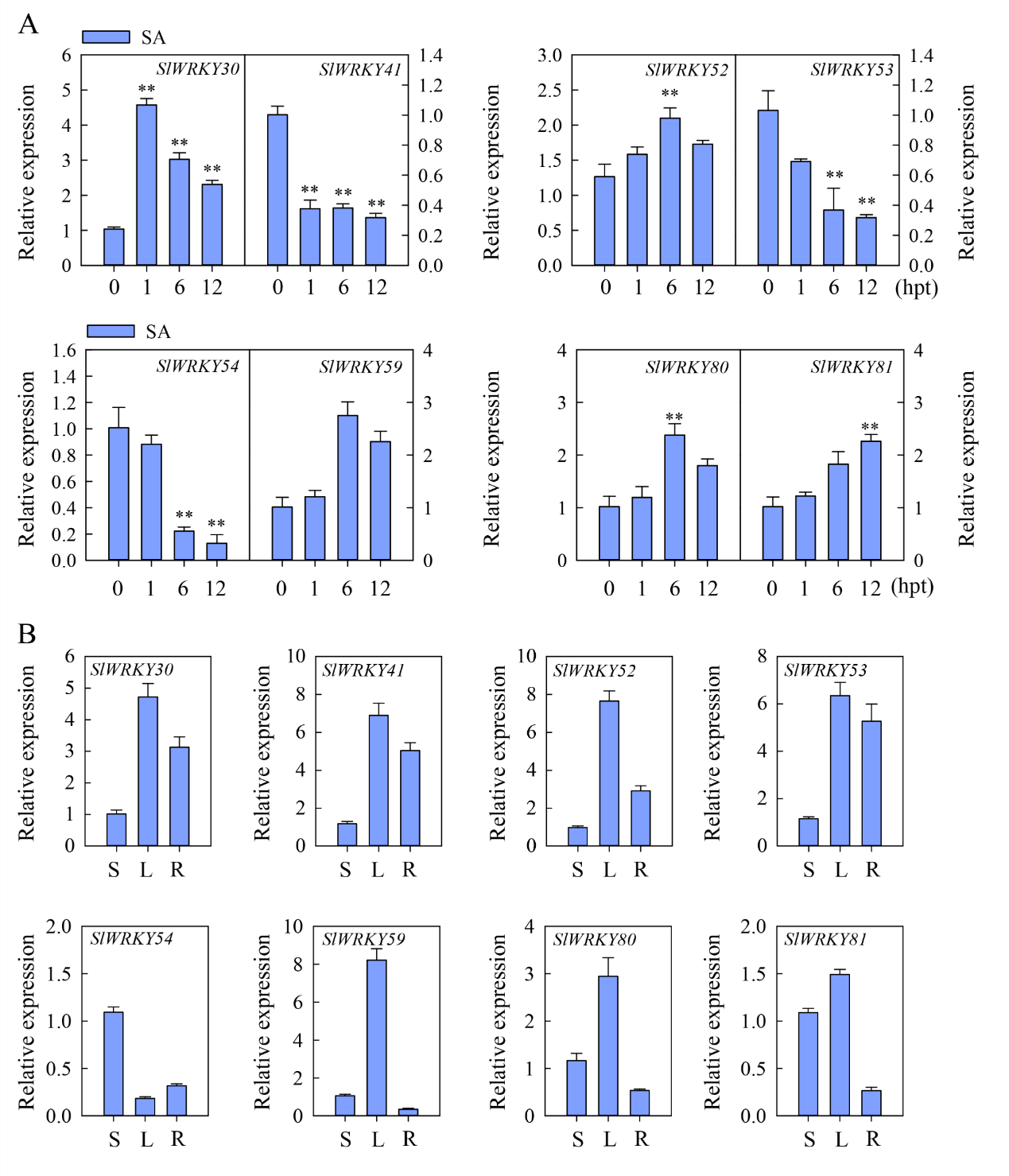
**Supplemental Figure 1. Expression analysis of the eight group Ⅲ *SlWRKY* genes induced by exogenous application of SA in tomato*.***

A, Expression levels of the eight group Ⅲ *SlWRKY* genes in tomato leaves analyzed by RT-qPCR at 0, 1, 6, and 12 hours after treatment with 200 μM SA. Data represent the mean ± SE.

B, Expression levels of the eight group Ⅲ *SlWRKY* genes analyzed by RT-qPCR in different tissues of four-week-old tomato seedlings. The relative expression of the genes in the leaf (L) and root (R) was compared with that in the stem (S), which was set to 1.


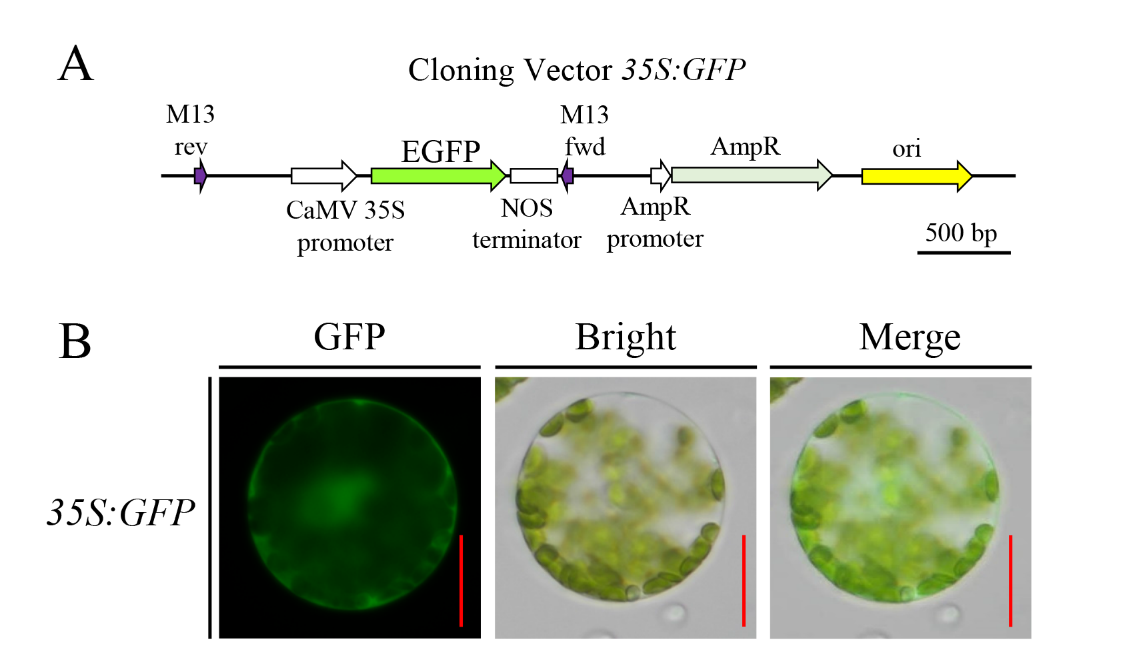


**Supplemental Figure 2. Subcellular localization of GFP protein in *Arabidopsis* mesophyll protoplasts.**

A, Schematic diagram of *35S:GFP* expression vector.

B, Subcellular localization of GFP protein.


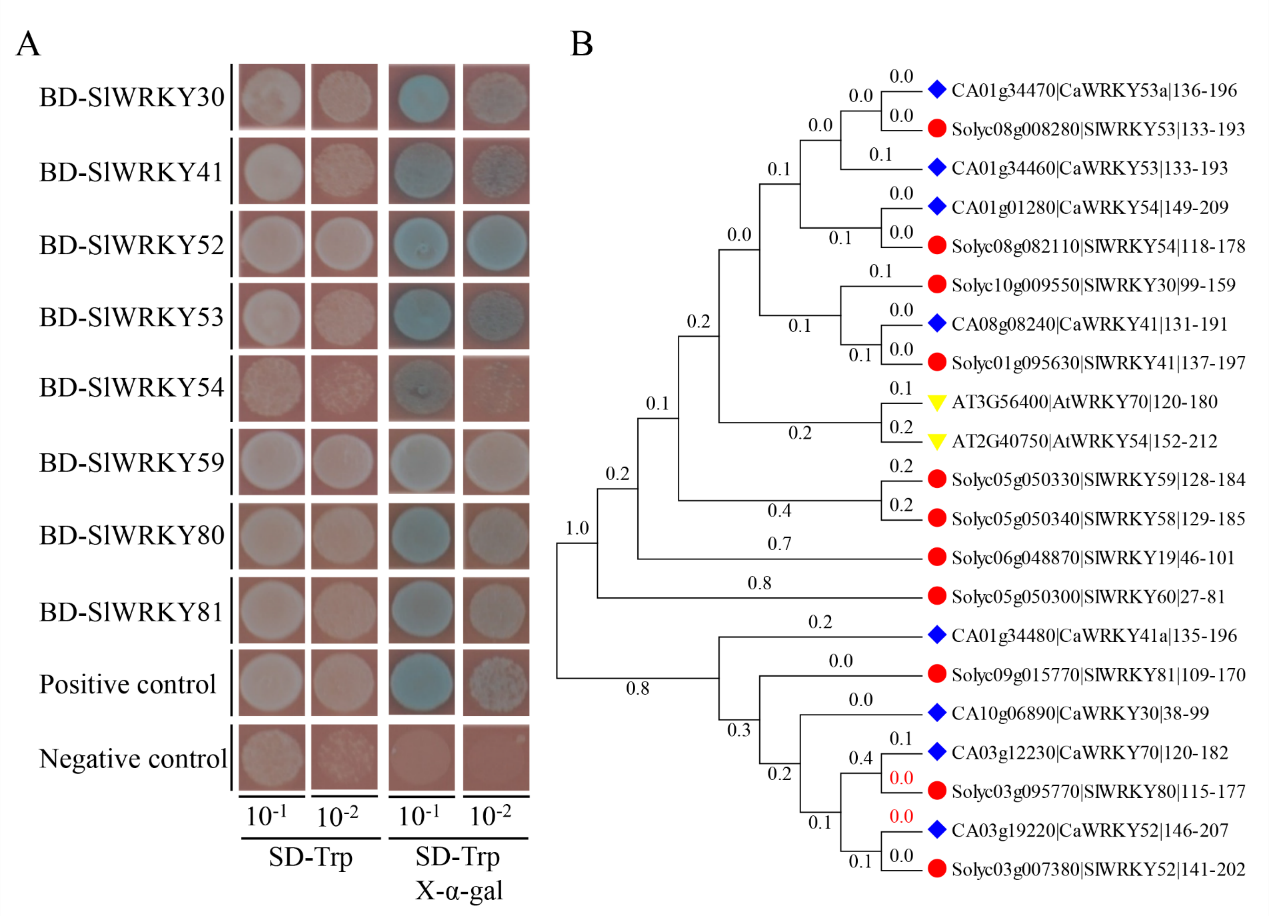


**Supplemental Figure 3. Transcriptional activation activity of the group Ⅲ SlWRKY members in yeast cells.**

A, Transcriptional activation assay of the group III SlWRKY members in yeast cells. *LacZ* reporter gene expression is indicated by blue color.

B, Phylogenetic tree for the group Ⅲ WRKY domains from tomato, pepper, and Arabidopsis. Amino acid sequences labeled with red circles, dark blue diamonds, and inverted yellow triangles represent the group Ⅲ WRKY domains from tomato, pepper and Arabidopsis, respectively.


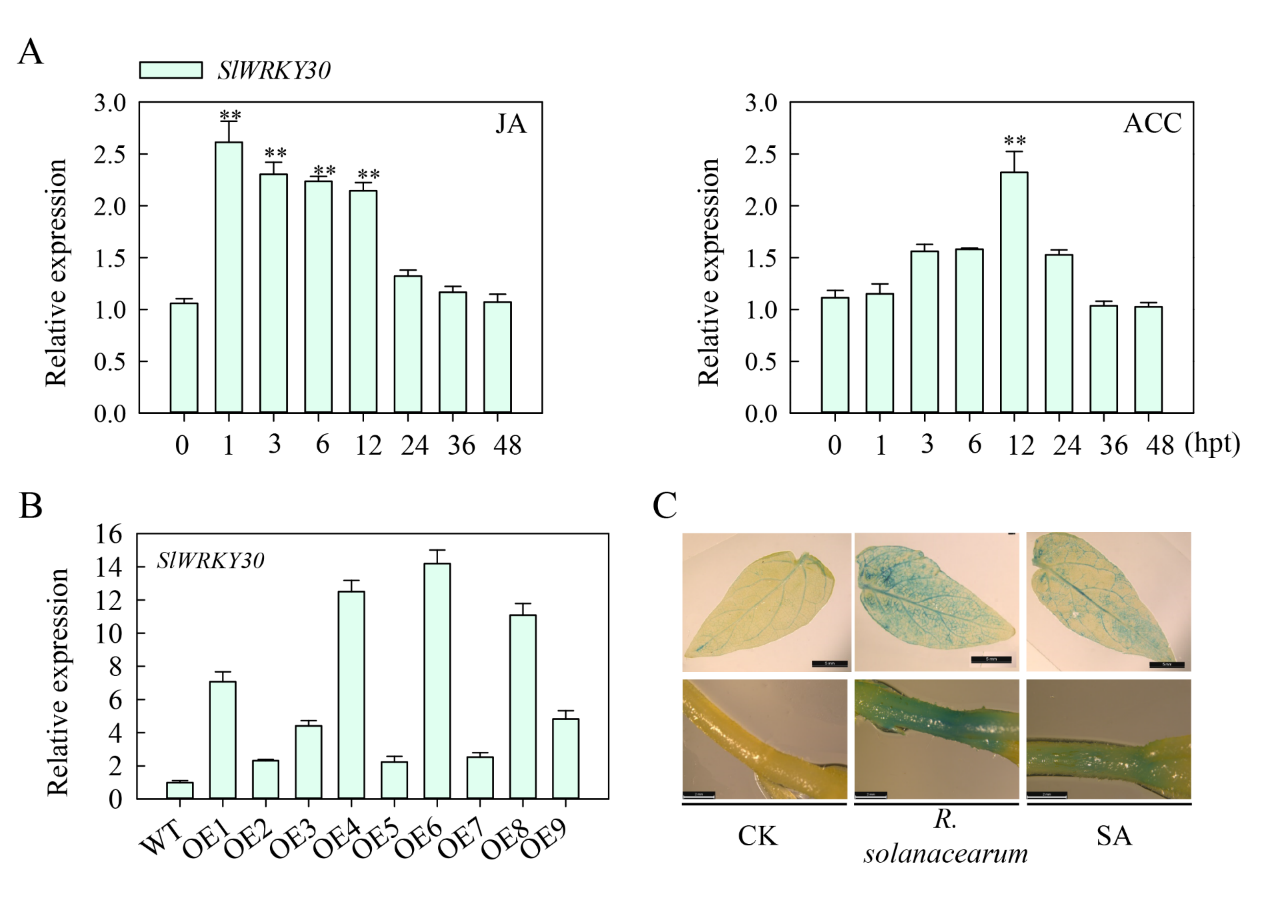


**Supplemental Figure 4. Expression analysis of *SlWRKY30* in tomato plants.**

A, Expression levels of *SlWRKY30* in tomato leaves analyzed by RT-qPCR at 0, 1, 3, 6, 12, 24, 36, and 48 h post treatment (hpt) with JA (jasmonic acid, 100 μM) and ACC (ethylene precursor, 1 μM).

B, RT-qPCR analysis of *SlWRKY30* expression in *SlWRKY30*-OE tomato lines.

C, GUS expression in in *pSlWRKY30:GUS* tomato.


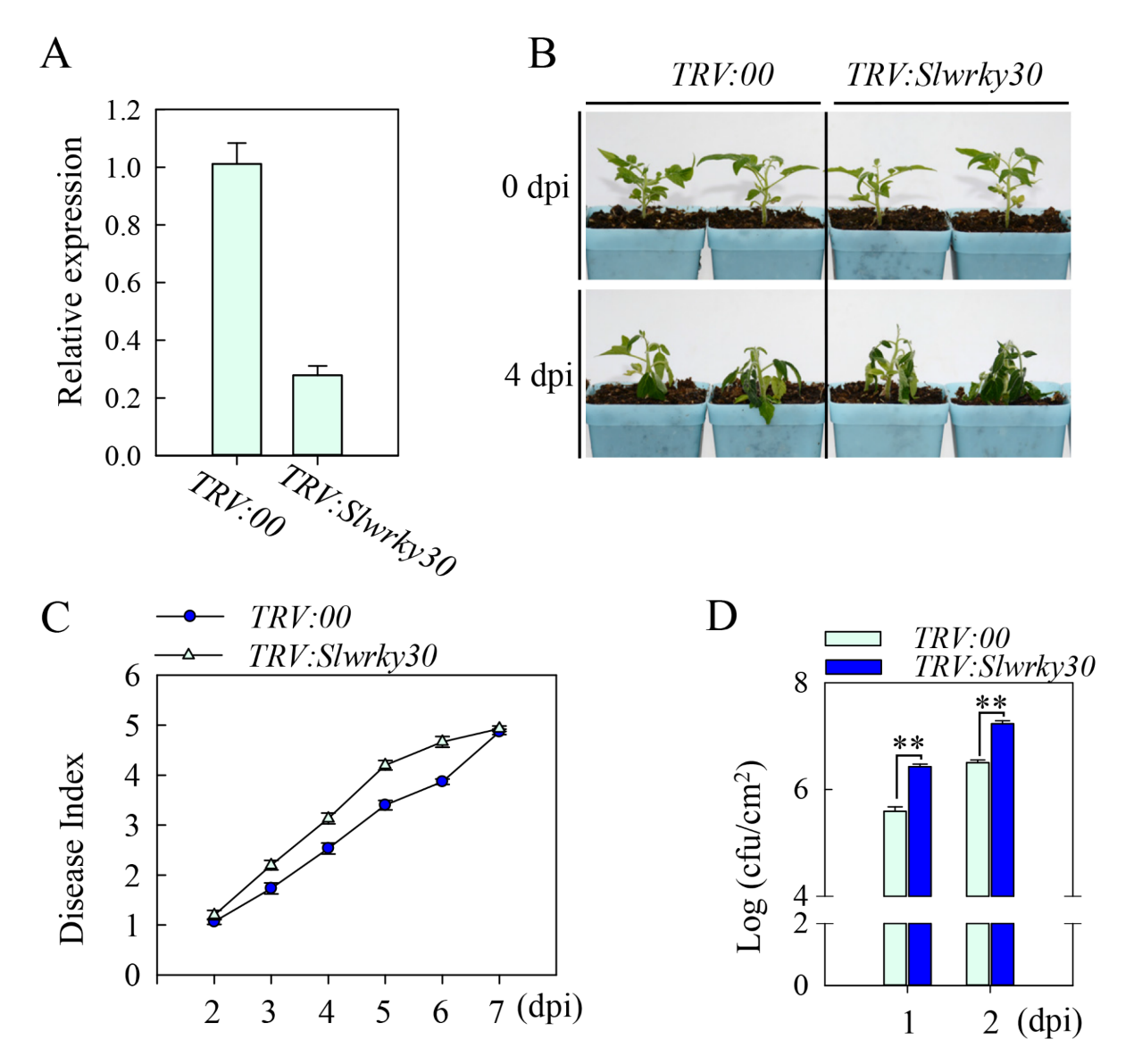


**Supplemental Figure 5. Silencing of *SlWRKY30* decreases tomato resistance to RSI.**

A, RT-qPCR analysis of *SlWRKY30* expression in *SlWRKY30-*silenced tomato plants.

B, Resistance levels in *TRV:Slwrky30* and *TRV:00* (empty vector control) tomato plants at 0 and 4 days post inoculation (dpi) with *R. solanacearum*.

C and D, Disease index (C) and bacterial growth (D) in *TRV:Slwrky30* and *TRV:00* tomato plants following RSI*.* Data represent the mean ± SE.


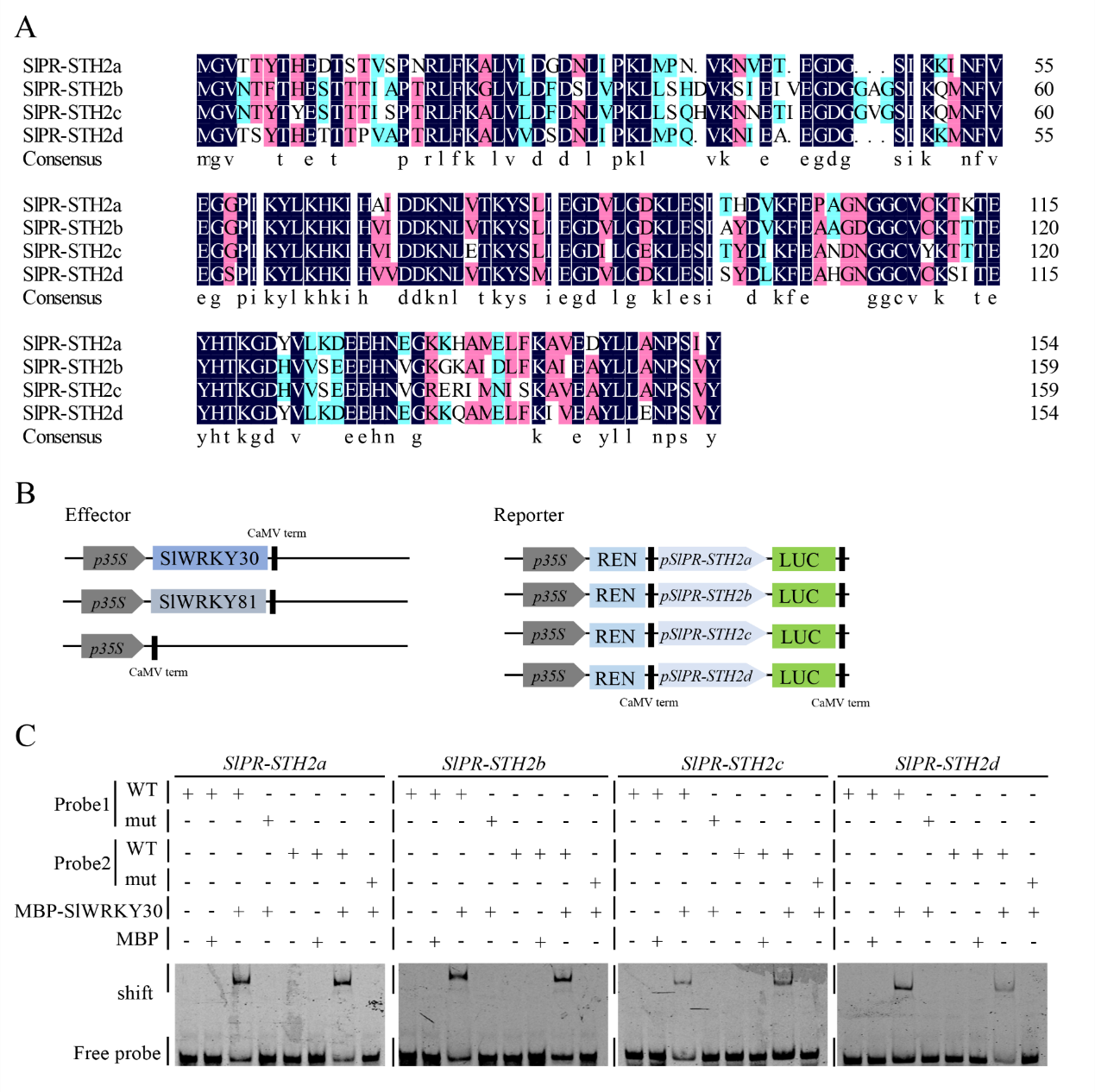


**Supplemental Figure 6. SlWRKY30 directly binds *SlPR-STH2a*, *SlPR-STH2b*, *SlPR­STH2c*, and *SlPR-STH2d* promoters.**

A, Alignment of deduced amino acid sequences of SlPR-STH2a, SlPR-STH2b, SlPR-STH2c, and SlPR­STH2d in tomato.

B, Structural schematic diagrams of the effector (*pGreenⅡ 62*­*SK*) and reporter (*pGreenⅡ*­*0800*­*LUC*) constructs that were used for the dual-luciferase assay. LUC: Firefly luciferase, REN: *Renilla* luciferase.

C, EMSA showing that SlWRKY30 directly binds to the *SlPR-STH2a/b/c/d* promoters.


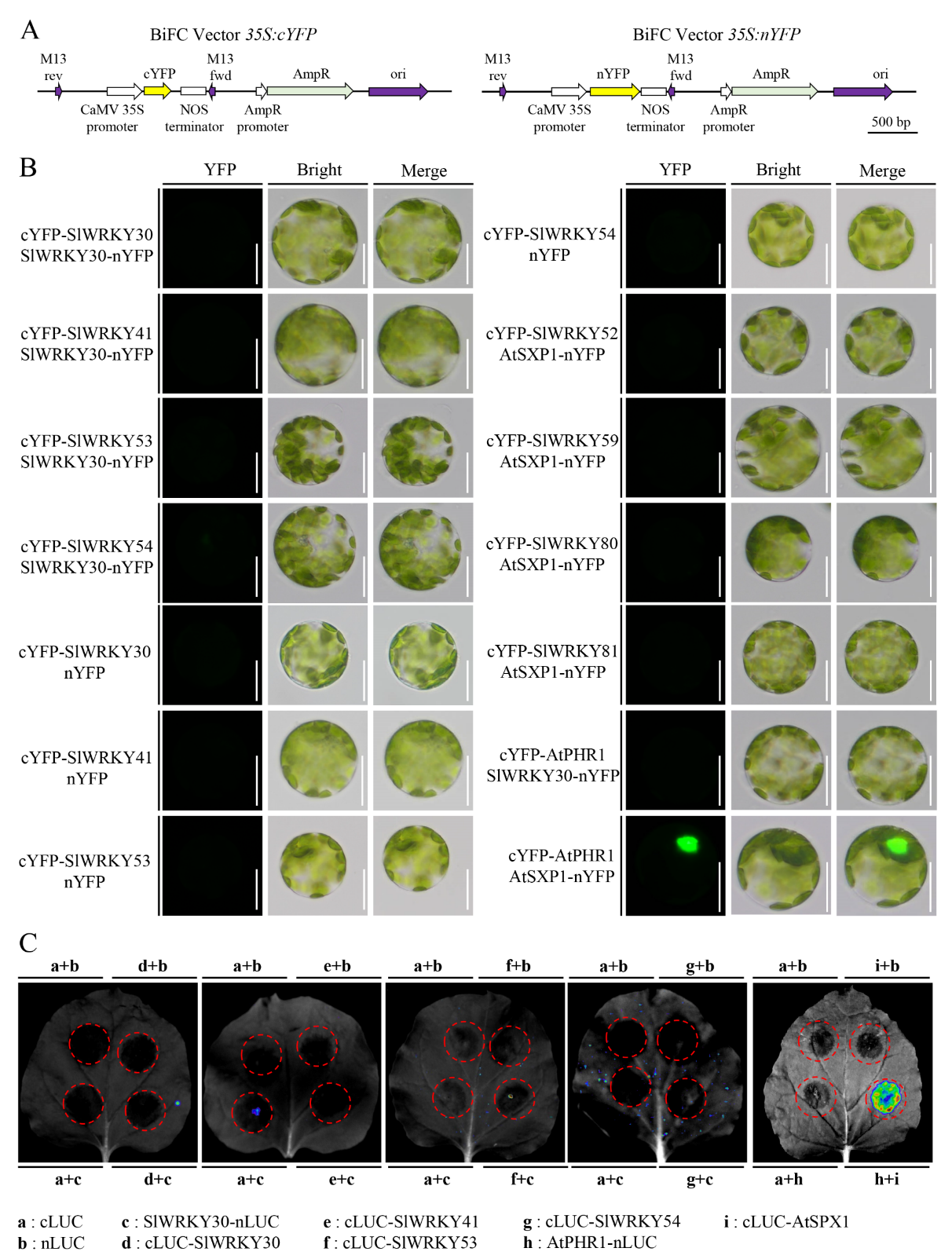


**Supplemental Figure 7. SlWRKY30 did not interact with SlWRKY30, SlWRKY41, SlWRKY53, and SlWRKY54.**

A, Schematic diagram of 35S:cYFP and 35S:nYFP BiFC vectors.

B, BiFC assay showing the interactions between SlWRKY30 and SlWRKY30, 41, 53, and 54 in Arabidopsis protoplasts. Bar=20 μm.

C, LCI assay showing no interaction between SlWRKY30 and SlWRKY30, 41, 53, and 54 in *N.benthamiana* leaves.

The interaction between AtSPX1 and AtPHR1 proteins was used as a positive control.


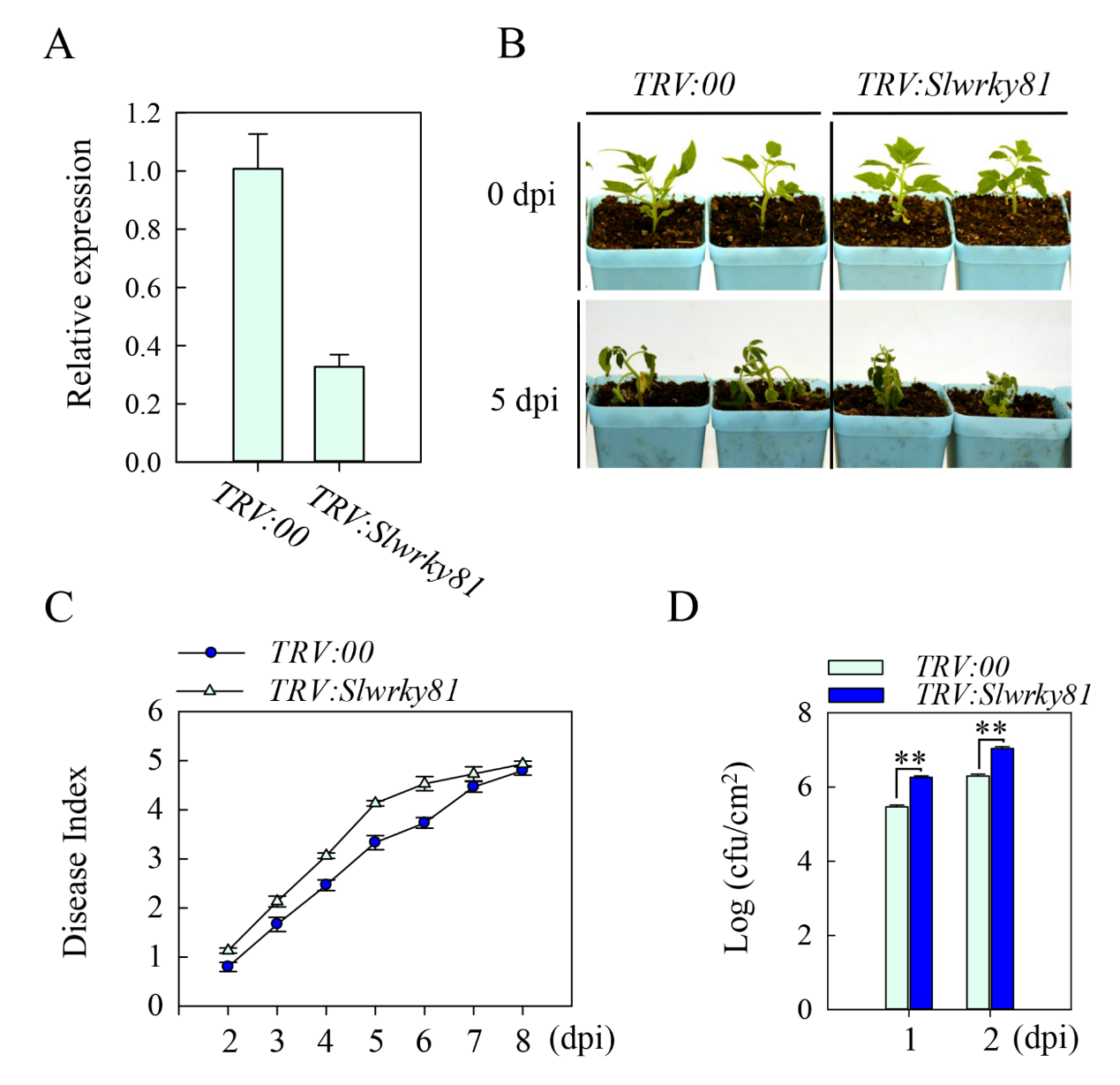


**Supplemental Figure 8. Silencing of *SlWRKY81* decreases tomato resistance to RSI.**

A, RT-qPCR analysis of *SlWRKY81* expression in *SlWRKY81-*silenced tomato plants.

B, Resistance levels in *TRV:Slwrky81* and *TRV:00* (empty vector control) tomato plants at 0 and 5 days post inoculation (dpi) with *R. solanacearum*.

C and D, Disease index (C) and bacterial growth (D) in *TRV:Slwrky81* and *TRV:00* tomato plants following RSI*.* Data represent the mean ± SE.


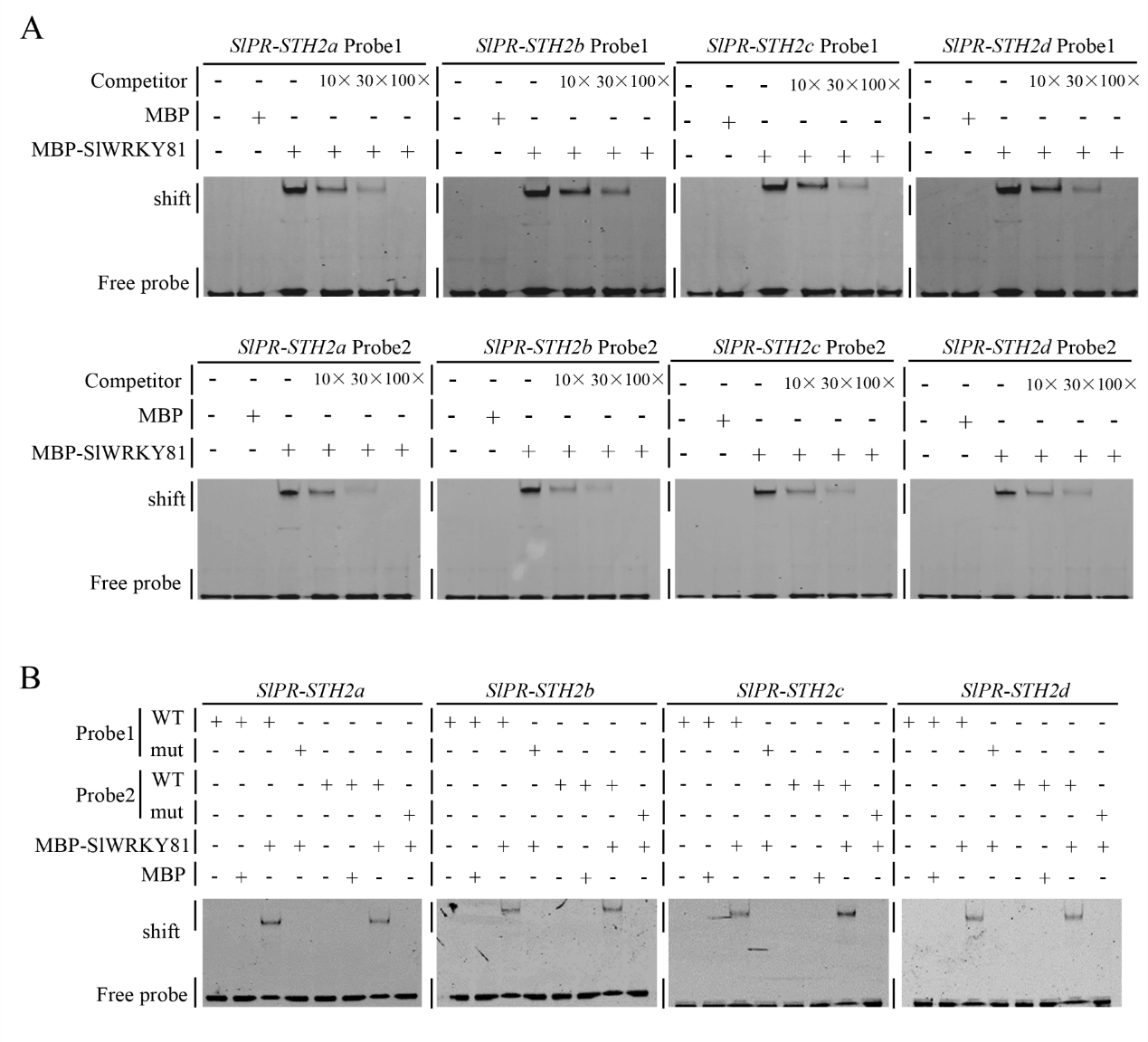


**Supplemental Figure 9. SlWRKY81 directly binds to the *SlPR-STH2a/b/c/d* promoters.**

A and B, EMSA showing that SlWRKY81 directly binds to the DNA Probes (generated from the *SlPR­STH2a/b/c/d* promoters).
